# Compatibility of Drinfeld presentations for split affine Kac–Moody quantum symmetric pairs

**DOI:** 10.1007/s11005-025-01964-7

**Published:** 2025-06-28

**Authors:** Jian-Rong Li, Tomasz Przeździecki

**Affiliations:** 1 Faculty of Mathematics, University of Vienna, Oskar Morgenstern Platz 1, 1090 Vienna, Austria; 2 School of Mathematics, University of Edinburgh, Peter Guthrie Tait Rd, Edinburgh, EH9 3FD United Kingdom

**Keywords:** Quantum symmetric pairs, Coideal subalgebras, $\imath$quantum groups, Kac–Moody algebras, *q*-Onsager algebra, Drinfeld presentation, Drinfeld polynomials, Braid group action, 17B37, 17B67, 81R10

## Abstract

Let $(\textbf{U}, \textbf{U}^\imath )$ be a split affine quantum symmetric pair of type $\textsf{B}_n^{(1)}, \textsf{C}_n^{(1)}$ or $\textsf{D}_n^{(1)}$. We prove factorization and coproduct formulae for the Drinfeld–Cartan operators $\Theta _i(z)$ in the Lu–Wang Drinfeld-type presentation, generalizing the type $\textsf{A}_n^{(1)}$ result from Przeździecki (arXiv:2311.13705). As an application, we show that a boundary analogue of the *q*-character map, defined via the spectra of these operators, is compatible with the usual *q*-character map. As an auxiliary result, we also produce explicit reduced expressions for the fundamental weights in the extended affine Weyl groups of classical types.

## Introduction

Quantum affine algebras admit three distinct presentations: the ‘Drinfeld–Jimbo’, ‘new Drinfeld’ and ‘RTT’ realizations. The first can be seen as a quantization of the usual Serre-type presentation of a Kac–Moody Lie algebra $\widehat{{\mathfrak {g}}}$, while the second is a quantization of the central extension presentation of $\widehat{{\mathfrak {g}}}$. The interplay between these different realizations was studied and described precisely in [13, 18]. One of the most interesting features of the new Drinfeld presentation is that it exhibits a large, infinitely generated, commutative subalgebra of $U_q(L{\mathfrak {g}})$. The spectra of the generators of this subalgebra, often called Drinfeld–Cartan operators $\phi ^\pm _{i,m}$, play a key role in the classification of finite-dimensional representation of $U_q(L{\mathfrak {g}})$ via Drinfeld polynomials [10, 11], and *q*-character theory [17, 19].

Recently, Lu and Wang [29], building on the work of Baseilhac and Kolb [6] in rank one (see also [31, 34]), have constructed a Drinfeld-type presentation for split and certain quasi-split affine quantum symmetric pair coideal subalgebras. The Lu–Wang presentation also exhibits a large commutative subalgebra, generated by $\Theta _{i,m}$. This very significant development raises many questions. Here, we focus on the following three. *Firstly*, what is the relationship between the generators $\Theta _{i,m}$ and the usual Drinfeld–Cartan operators $\phi ^\pm _{i,m}$? *Secondly*, how to describe the coproduct of $\Theta _{i,m}$? *Thirdly*, can the Lu–Wang presentation be used to define a ‘boundary’ analogue of *q*-characters?

The first two questions were answered by the second author [32] for split affine quantum symmetric pairs of type $\textsf{A}_n^{(1)}$. In particular, it was shown that the generating series $\pmb {\grave{\Theta }}_i(z)$ have the following *factorization property*:

1.1 $$\begin{aligned} \pmb {\grave{\Theta }}_i(z) \equiv \pmb {\phi }_i^-(z^{-1})\pmb {\phi }_i^+(C z) \quad \mod {U_q(L{\mathfrak {g}})_+}[\negthinspace [z]\negthinspace ], \end{aligned}$$

where $U_q(L{\mathfrak {g}})_+$ is the Drinfeld positive half of $U_q(L{\mathfrak {g}})$. Moreover, the series $\pmb {\grave{\Theta }}_i(z)$ are *approximately group-like*, in the sense that

1.2 $$\begin{aligned} \Delta (\pmb {\grave{\Theta }}_i(z)) \equiv \pmb {\grave{\Theta }}_i(z) \otimes \pmb {\grave{\Theta }}_i(z) \quad \mod {U_q(L{\mathfrak {g}}) \otimes U_q(L{\mathfrak {g}})_{+}}[\negthinspace [z]\negthinspace ]. \end{aligned}$$

The main result of the present paper is a generalization of the two results above to split affine quantum symmetric pairs of types $\textsf{B}_n^{(1)}, \textsf{C}_n^{(1)}$ and $\textsf{D}_n^{(1)}$. As an application, we also propose a definition of boundary *q*-characters and prove that they are, in a suitable sense, compatible with the usual *q*-characters.

### Proof strategy

Our proof is based on an algebraic and computational approach, using the Drinfeld–Jimbo and new Drinfeld presentations only. In particular, we do not use any information available through the RTT presentation. Since the overall proof is quite technical, let us summarize the main steps below.

*Step 1: compute explicit reduced expressions for the fundamental weights* $\omega _i$. This is carried out in Sect. 3. Whenever $\alpha _i$ has multiplicity one in the highest root, the expressions can be derived from a formula in [7]. In the remaining cases, we consider the extended affine Weyl group as a group of affine transformations of ${\mathbb R}^n$ and compute that our expressions act as the correct affine shifts. To show that they are reduced, we calculate their lengths and compare them with a formula from [20].

*Step 2: express* $\textbf{T}_{\omega ^{\prime }_i}(B_i)$ *as an explicit polynomial in the variables* $B_j$. As in the usual Drinfeld presentation, the generators in the Lu–Wang presentation are constructed using a braid group action (which differs from the ordinary Lusztig action). More precisely, they are constructed by repeatedly applying the braid group operators $\textbf{T}_{\omega _i}$ to the usual Kolb–Letzter generators of the coideal subalgebra. Using the explicit formulae for $\omega _i$ from Step 1, we are able to express $\textbf{T}_{\omega ^{\prime }_i}(B_i)$ by means of certain recursively defined polynomials in the variables $B_j$. This is carried out in Sect. 7, based on the auxiliary calculations from Sect. 4.

*Step 3: show that* $\textbf{T}_{\omega ^{\prime }_i}(B_i) \equiv T_{\omega ^{\prime }_i}(B_i)$. More precisely, we show that the Lu–Wang braid group operator $\textbf{T}_{\omega ^{\prime }_i}$ coincides with the usual Lusztig braid group operator $T_{\omega ^{\prime }_i}$ on $B_i$, modulo a certain subalgebra of $U_q(L{\mathfrak {g}})_+$. This is achieved in Sect. 7 (Theorem 7.1) in the following way. Recall that $B_j$ is a linear combination of $F_j$ and $E_jK_j^{-1}$. Writing $\textbf{T}_{\omega ^{\prime }_i}(B_i)$ as a polynomial as in Step 2, and substituting for each occurrence of $B_j$ either $F_j$ or $E_jK_j^{-1}$, we can express $\textbf{T}_{\omega ^{\prime }_i}(B_i)$ as a sum of homogeneous terms in the Drinfeld gradation. The main problem is to show that, except for $T_{\omega ^{\prime }_i}(E_iK_i^{-1})$, the terms which are not in $U_q(L{\mathfrak {g}})_+$ vanish. This is solved using the theory of good polynomials developed in Sect. 5, with the aid of auxiliary calculations from Sect. 6.

*Step 4: deduce the factorization and coproduct formulae.* The main idea is that, modulo $U_q(L{\mathfrak {g}})_+$, we can reduce the problem to rank one subalgebras and apply the results from [32]. We show that, to perform this reduction, we only need to verify a single commutation condition. This criterion is then checked explicitly in type $\textsf{D}_n^{(1)}$. The same methods carry over to types $\textsf{B}_n^{(1)}$ and $\textsf{C}_n^{(1)}$. This is achieved in Sect. 8.

We remark that the overall proof strategy is similar to that in [32, §9]. However, each step of the proof is significantly harder than in type $\textsf{A}$ case, mainly due to the higher complexity of reduced expressions for fundamental weights in other classical types. Another innovation is the concept of ‘good polynomials’, which allows us to systematically handle different types, and is also likely to find application in, e.g., the setting of quasi-split affine quantum symmetric pairs.

### Reduced expressions for fundamental weights

In Step 1 above, we compute explicit reduced expressions for the fundamental weights in the extended affine Weyl groups of classical types. Somewhat surprisingly, to our knowledge, such explicit expressions have not appeared in the literature before.^1^ As this result is interesting in its own right, we collect the expressions in a list below for quick reference, see Table 1.

**Table 1:** Reduced expressions for fundamental weights $\omega _i$ in extended affine Weyl groups of classical type

Type	Fundamental weight $\omega _i$	Range of *i*
$\textsf{A}_n^{(1)}$		$1 \le i \le n$
$\textsf{B}_n^{(1)}$		$1 \le i \le n$, *i* is odd
		$2 \le i \le n$, *i* is even
$\textsf{C}_n^{(1)}$		$i=n$
		$$i<n$$
$\textsf{D}_n^{(1)}$		, *i* is even
		, *i* is even
		$i=n$, *i* is even
		, *i* is odd
		, *i* is odd
		$i=n$, *i* is odd

Here $$[k,l] = s_k s_{k+1} \cdots s_{l}$$ and

### Application to *q*-characters

The notion of *q*-characters can be defined in at least three equivalent ways: via the universal *R*-matrix, via the spectrum of Drinfeld–Cartan operators, or via Nakajima’s quiver varieties. The quantum symmetric pair analogues of these approaches are objects of intense study, see, e.g., [1–3] for the latest developments on the universal *K*-matrix, and [24] for a quiver variety approach to symmetric pairs. Nevertheless, it appears that the geometric and integrable systems methods are not yet advanced enough to yield a satisfactory theory of *q*-characters for quantum symmetric pairs. The main obstacle in the latter case is the unavailability of a Khoroshkin–Tolstoy–Levendorsky–Soibelman–Stukopkin–Damiani-type factorization of the (affine) universal *K*-matrix.

In light of the aforementioned difficulties, we propose to define boundary *q*-characters directly via the Lu–Wang presentation instead. More precisely, we consider the generalized eigenspace decomposition of a finite-dimensional representation with respect to the action of the operators $\Theta _{i,m}$, and let boundary *q*-characters encode the multiplicities of such eigenspaces. This is equivalent to taking the trace of a certain operator in the completion ${\textbf{U}^\imath \otimes U_q(\widetilde{\mathfrak {h}})}[\negthinspace [z]\negthinspace ]$, yielding a map $\chi _q^\imath :\operatorname {Rep}\textbf{U}^\imath \rightarrow {U_q(\widetilde{\mathfrak {h}})}[\negthinspace [z]\negthinspace ]$, where $U_q(\widetilde{\mathfrak {h}}) = \langle k_i, h_{i,r} \mid i \in \mathbb {I}_0, r \le 0 \rangle$ is a ‘half’ of the Drinfeld–Cartan subalgebra of $U_q(L{\mathfrak {g}})$. We apply (1.1)–(1.2) to show that $\chi _q^\imath$ is compatible with the usual *q*-character map (Corollary 9.3). More precisely, we show that the diagram 
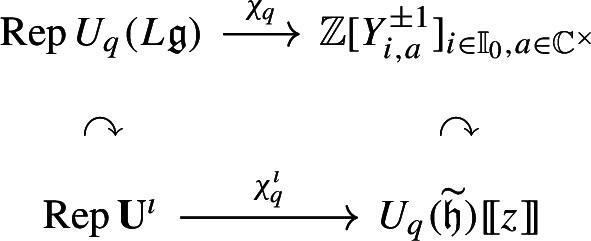
 commutes if ${U_q(\widetilde{\mathfrak {h}})}[\negthinspace [z]\negthinspace ]$ is endowed with an appropriate ‘twisting’ action of ${\mathbb Z}[Y_{i,a}^{\pm 1}]_{i \in \mathbb {I}_0, a \in {\mathbb C}^{\times }}$. In particular, this result yields an easy way to compute the boundary *q*-characters of restriction representations. For first results in the case of more general representations, we refer the reader to [25].

## Preliminaries

We work over the field of complex numbers and assume that $q \in {\mathbb C}^\times$ is not a root of unity throughout.

### Quantum affine algebras

Let $\mathbb {I}_0 = \{ 1,\cdots , n \}$ and $\mathbb {I}= \mathbb {I}_0 \cup \{0\}$. Let ${\mathfrak {g}}$ be a simple Lie algebra with Cartan matrix $(a_{ij})_{i,j \in \mathbb {I}_0}$, and $\widehat{{\mathfrak {g}}}$ the corresponding untwisted affine Lie algebra with affine Cartan matrix $(a_{ij})_{i,j \in \mathbb {I}}$. Let $d_i$ be relatively prime positive integers such that $(d_ia_{ji})_{i,j\in \mathbb {I}_0}$ is a symmetric matrix, and set $q_i = q^{d_i}$. We use Bourbaki’s conventions [7] for the Cartan matrix in non-simply laced types, i.e.,

$$\begin{aligned} \left( \begin{array}{rrrrrrr} 2 &  -1 &  \hdots &  0 &  0 \\ -1 &  2 &  \hdots &  0 &  0 \\ \vdots &  \vdots &  \ddots &  \vdots &  \vdots \\ 0 &  0 &  \hdots &  2 &  -2 \\ 0 &  0 &  \hdots &  -1 &  2 \end{array}\right) \quad , \quad \left( \begin{array}{rrrrrrr} 2 &  -1 &  \hdots &  0 &  0 \\ -1 &  2 &  \hdots &  0 &  0 \\ \vdots &  \vdots &  \ddots &  \vdots &  \vdots \\ 0 &  0 &  \hdots &  2 &  -1 \\ 0 &  0 &  \hdots &  -2 &  2 \end{array}\right) \end{aligned}$$

are the Cartan matrices of types $\textsf{B}_n$ and $\textsf{C}_n$, respectively.

We use standard notations and conventions regarding root systems, weight lattices, Weyl groups, etc., as in, e.g., [29, §3.1]. In particular, we let $\alpha _i$ ($i \in \mathbb {I}$) denote the simple roots of $\widehat{{\mathfrak {g}}}$; let $\theta$ denote the highest root of ${\mathfrak {g}}$, and $\delta$ the basic imaginary root; let *P* and *Q* denote the weight and root lattices of ${\mathfrak {g}}$, respectively; and let $\omega _i \in P$ ($i \in \mathbb {I}_0$) be the fundamental weights of ${\mathfrak {g}}$. Note that, according to Bourbaki’s conventions, $s_{\alpha _i}(\alpha _j) = \alpha _j - a_{ji} \alpha _i$.

The *quantum affine algebra* $U_q(\widehat{\mathfrak {g}})$ is the algebra with generators $e_i^\pm , K_i^{\pm 1}$ $(i \in \mathbb {I})$ and relations:

2.1 $$\begin{aligned} K_iK_i^{-1}&= K_i^{-1}K_i = 1, \nonumber \\ K_iK_j&= K_j K_i, \nonumber \\ K_ie_j^{\pm }&= q_i^{\pm a_{ji}} e_j^\pm K_i, \nonumber \\ {EMPTY}[e_i^{+}, e_j^{-}]&= \delta _{ij} \frac{K_i - K_i^{-1}}{q_i - q_i^{-1}}, \end{aligned}$$

2.2 $$\begin{aligned} \textsf{Serre}_{ij}(e_i^{\pm }, e_j^\pm )&= 0 \qquad (i \ne j), \end{aligned}$$

whereWe will also sometimes abbreviate

$$ E_i = e_i^+, \qquad F_i = e_i^-. $$

We use the standard notation for divided powers, i.e., $(e^{\pm }_i)^{(r)} = (e^{\pm }_i)^{r}/[r]_{q_i}!$. Given $\mu = \sum _{i \in \mathbb {I}} c_i \alpha _i \in {\mathbb Z}\mathbb {I}= \bigoplus _{i \in \mathbb {I}} {\mathbb Z}\alpha _i$, set

2.3 $$\begin{aligned} K_\mu = \textstyle \prod _{i \in \mathbb {I}} K_i^{c_i}, \qquad K_{\delta } = K_0 K_{\theta }. \end{aligned}$$

The algebra $U_q(\widehat{\mathfrak {g}})$ is a Hopf algebra, with the coproduct

$$\begin{aligned} \Delta (e_i^+) = e_i^+ \otimes 1 + K_i \otimes e_i, \quad \Delta (e_i^-) = e_i^- \otimes K_i^{-1} + 1 \otimes e_i^-, \quad \Delta (K_i^{\pm 1}) = K_i^{\pm 1} \otimes K_i^{\pm 1}. \end{aligned}$$

The counit is given by

$$ \varepsilon (e_i^{\pm }) = 0, \quad \varepsilon (K_i^{\pm 1}) = 1. $$

The *quantum loop algebra* $U_q(L\mathfrak {g})$ is the quotient of $U_q(\widehat{\mathfrak {g}})$ by the ideal generated by the central element $K_\delta - 1$. Let us recall the “new" Drinfeld presentation of the quantum loop algebra $U_q(L\mathfrak {g})$. By [4, 16], $U_q(L\mathfrak {g})$ is isomorphic to the algebra generated by $x^{\pm }_{i,k}, h_{i,l}, K^{\pm 1}_{i}$, where $k \in {\mathbb Z}$, $l \in {\mathbb Z}- \{0\}$ and $i \in \mathbb {I}_0$, subject to the following relations:where $\operatorname {Sym}_{k_1,\cdots ,k_r}$ denotes symmetrization with respect to the indices $k_1, \cdots , k_r$ and

$$ \pmb {\phi }^{\pm }_i(z) = \sum _{k=0}^\infty \phi ^\pm _{i,\pm k} z^{\pm k} = K_i^{\pm 1} \exp \left( \pm (q-q^{-1}) \sum _{k=1}^\infty h_{i,\pm k} z^{\pm k} \right) . $$

Let

$$ {\widetilde{U}}_q(L\mathfrak {g})= U_q(L\mathfrak {g})\otimes {\mathbb C}[\mathbb {K}_i^{\pm 1} \mid i \in \mathbb {I}]. $$

It can be regarded as a version of the Drinfeld double of $U_q(\widehat{\mathfrak {n}}_+)$. Given $\mu \in {\mathbb Z}\mathbb {I}$, we define $\mathbb {K}_\mu$ in the same way as in (2.3). We consider ${\widetilde{U}}_q(L\mathfrak {g})$ as a *Q*-graded algebra with

$$ \operatorname {deg}^{\textrm{Dr}}x^{\pm }_{i,k} = \pm \alpha _i, \quad \operatorname {deg}^{\textrm{Dr}}h_{i,k} = \operatorname {deg}^{\textrm{Dr}}\mathbb {K}_i = 0 \qquad (i \in \mathbb {I}_0). $$

In the following, we will use the abbreviations

$$ \textbf{U}= U_q(L\mathfrak {g}), \qquad \widetilde{\textbf{U}}= {\widetilde{U}}_q(L\mathfrak {g}). $$

Let $\widetilde{\textbf{U}}_+$ be the subalgebra of $\widetilde{\textbf{U}}$ spanned by elements of positive degree, i.e., nonnegative along all $\alpha _i$, and positive along at least one $\alpha _i$ ($i \in \mathbb {I}_0$).

We will also need to consider other subalgebras of $\widetilde{\textbf{U}}$ spanned by elements of specified degree. Write $\operatorname {deg}^{\textrm{Dr}}_i$ for degree along the simple root $\alpha _i$. Let $Q_i$ be the sublattice of *Q* spanned by all the simple roots except $\alpha _i$. Let $\widetilde{\textbf{U}}_{d_i=r,+}$ be the subalgebra of $\widetilde{\textbf{U}}$ spanned by elements which are both: (i) of degree *r* along $\alpha _i$ and (ii) positive $Q_i$-degree, i.e., nonnegative along all the simple roots and positive along at least one simple root in $Q_i$. That is,

$$ \widetilde{\textbf{U}}_{d_i=r,+} {=} \langle y \in \widetilde{\textbf{U}}\mid \operatorname {deg}^{\textrm{Dr}}_i y = r;\ \forall j \ne i \in \mathbb {I}_0:\operatorname {deg}^{\textrm{Dr}}_j y \ge 0;\ \exists j \ne i:\operatorname {deg}^{\textrm{Dr}}_j y {>} 0 \rangle . $$

Moreover, set

$$\begin{aligned} \widetilde{\textbf{U}}_{d_i\ge r,+} =&\ \langle y \in \widetilde{\textbf{U}}\mid \operatorname {deg}^{\textrm{Dr}}_i y \ge r;\ \forall j \ne i \in \mathbb {I}_0:\operatorname {deg}^{\textrm{Dr}}_j y \ge 0;\ \exists j \ne i:\operatorname {deg}^{\textrm{Dr}}_j y> 0 \rangle , \\ \widetilde{\textbf{U}}_{\ne i,+} =&\ \bigoplus _{r \in {\mathbb Z}} \widetilde{\textbf{U}}_{d_i=r,+} = \langle y \in \widetilde{\textbf{U}}\mid \forall j \ne i \in \mathbb {I}_0:\operatorname {deg}^{\textrm{Dr}}_j y \ge 0;\ \exists j \ne i:\operatorname {deg}^{\textrm{Dr}}_j y> 0 \rangle , \\ \widetilde{\textbf{U}}_+ =&\ \langle y \in \widetilde{\textbf{U}}\mid \forall j \in \mathbb {I}_0:\operatorname {deg}^{\textrm{Dr}}_j y \ge 0,\ \exists j :\operatorname {deg}^{\textrm{Dr}}_j y > 0 \rangle . \end{aligned}$$

We will also need the following lemma.

#### Lemma 2.1

We have

$$ \operatorname {deg}^{\textrm{Dr}}e_0^{\pm } = \mp \theta . $$

#### Proof

This follows directly from the explicit description of the images of $E_0$ and $F_0$ in the Drinfeld realization, see, e.g., [4, Remark 4.7] and [11, Theorem 2.2]. $\square$

### The braid group action

The Weyl group $W_0$ of ${\mathfrak {g}}$ is generated by the simple reflections $s_i$ ($i \in \mathbb {I}_0$), and acts on *P* in the usual way. The extended affine Weyl group ${\widetilde{W}}$ is the semi-direct product $W_0 \ltimes P$. It contains the affine Weyl group $W = W_0 \ltimes Q = \langle s_i \mid i \in \mathbb {I}\rangle$ as a subgroup and can also be realized as the semi-direct product

$$\begin{aligned} {\widetilde{W}} \cong \Lambda \ltimes W, \end{aligned}$$

where $\Lambda = P/Q$ is a finite group of automorphisms of the Dynkin diagram of $\widehat{{\mathfrak {g}}}$. Let $\widetilde{\mathcal {B}}$ be the braid group associated with ${\widetilde{W}}$.

It is well known [28, §37.1.3] that, for each $i \in \mathbb {I}$, there exists an automorphism $T_i$ of $U_q(L\mathfrak {g})$ such that $T_i(K_\mu ) = K_{s_i\mu }$ and

$$\begin{aligned} T_i(E_i) =&\ -F_iK_i, \quad \quad T_i(E_j) = \ \sum _{r+s = -a_{ji}} (-1)^r q_i^{-r} E_i^{(s)}E_jE_i^{(r)}, \\ T_i(F_i) =&\ -K_i^{-1}E_i, \quad \quad T_i(F_j) = \ \sum _{r+s = -a_{ji}} (-1)^r q_i^{r} F_i^{(r)}F_jF_i^{(s)}, \end{aligned}$$

for $\mu \in P$ and $i \ne j$. For each $\lambda \in \Lambda$, there is also an automorphism $T_\lambda$ such that $T_\lambda (E_i) = E_{\lambda (i)}$, $T_\lambda (F_i) = F_{\lambda (i)}$ and $T_\lambda (K_i) = K_{\lambda (i)}$. These automorphisms satisfy the relations of the braid group $\widetilde{\mathcal {B}}$. We extend this action to an action on $\widetilde{\textbf{U}}$ by setting $T_i(\mathbb {K}_\mu ) = \mathbb {K}_{s_i\mu }$ and $T_\lambda (\mathbb {K}_i) = \mathbb {K}_{\lambda (i)}$. Given a reduced expression $w = \lambda s_{i_1} \cdots s_{i_r} \in {\widetilde{W}}$, one defines $T_w = T_\lambda T_{i_1} \cdots T_{i_r}$. This is independent of the choice of reduced expression.

### Quantum symmetric pairs of split affine type

We recall the definition of quantum symmetric pair coideal subalgebras of split affine type, i.e., corresponding to Satake diagrams with no black nodes and no involution. More precisely, we consider a “universal” version of these algebras introduced in [30, 34], also known as universal $\imath$quantum groups.

#### Definition 2.2

Let $\widetilde{\textbf{U}}^\imath = \widetilde{\textbf{U}}^\imath (\widehat{{\mathfrak {g}}})$ be the algebra generated by $B_i$ and central invertible elements $\mathbb {K}_i$ $(i \in \mathbb {I})$ subject to relations

$$ -q_i \mathbb {K}_i^{-1}\textsf{Serre}_{ij}(B_i,B_j) = \left\{ \begin{array}{r l l} 0 &  \text{ if } &  a_{ji} = 0, \\ B_j &  \text{ if } &  a_{ji} = -1, \\ {[2]}_{q_i}^2[B_i, B_j] &  \text{ if } &  a_{ji} = -2, \\ (1+[3]_{q_i})(B_i^2B_j+B_jB_i^2) \\ - [4]_{q_i}(1+[2]_{q_i}^2)B_iB_jB_i + q_i^{-1}[3]_{q_i}^2\mathbb {K}_iB_j &  \text{ if } &  a_{ji} = -3. \end{array} \right. $$

It follows from [22, Theorem 8.3] that $Z(\widetilde{\textbf{U}}^\imath ) = {\mathbb C}[\mathbb {K}_i^{\pm 1} \mid i \in \mathbb {I}]$. Recently, Lu–Wang [29] and Zhang [34] found a “Drinfeld-type" presentation of $\widetilde{\textbf{U}}^\imath$.

#### Definition 2.3

Let  be the $\mathbb {C}$-algebra generated by $H_{i,m}$ and $A_{i,r}$, where $m\ge 1$, $r\in {\mathbb Z}$, and invertible central elements $\mathbb {K}_i$ ($i \in \mathbb {I}_0$), $\mathfrak {C}$, subject to the following relations:

$$\begin{aligned}&=0, \\ [H_{i,m}, A_{j,r}]&= \textstyle \frac{[m \cdot a_{ji}]_{q_i}}{m} (A_{j,r+m}- A_{j,r-m}\mathfrak {C}^m), \\ [A_{i,r}, A_{j,s}]&= 0 \quad \text{ if } \quad a_{ji} = 0, \\ [A_{i,r}, A_{j,s+1}]_{q_i^{-a_{ji}}} -q_i^{-a_{ji}} [A_{i,r+1}, A_{j,s}]_{q^{a_{ji}}}&= 0 \quad \text{ if } \quad i\ne j, \\ [A_{i,r}, A_{i,s+1}]_{q_i^{-2}} -q_i^{-2} [A_{i,r+1}, A_{i,s}]_{q_i^{2}}&= q_i^{-2}\mathbb {K}_i\mathfrak {C}^r\Theta _{i,s-r+1} - q_i^{-4}\mathbb {K}_i\mathfrak {C}^{r+1}\Theta _{i,s-r-1} \\&\quad + q_i^{-2}\mathbb {K}_i\mathfrak {C}^s\Theta _{i,r-s+1} -q_i^{-4}\mathbb {K}_i \mathfrak {C}^{s+1}\Theta _{i,r-s-1},\end{aligned}$$

and Serre relations (see [34, (3.27)–(3.28)]), where $m,n\ge 1$; $r,s, r_1, r_2\in {\mathbb Z}$ and

$$\begin{aligned} 1+ \sum _{m=1}^\infty (q-q^{-1})\Theta _{i,m} z^m = \exp \left( (q-q^{-1}) \sum _{m=1}^\infty H_{i,m} z^m \right) . \end{aligned}$$

By convention, $\Theta _{i,0} = (q-q^{-1})^{-1}$ and $\Theta _{i,m} = 0$ for $m\le -1$.

By [34, Theorem 3.4], there is an algebra isomorphismThis isomorphism and its inverse can be described in terms of the braid group action recalled in Sect. 2.4. For details, see [34, (3.1)–(3.3)]. In particular, it sends

$$\begin{aligned} B_{i} \mapsto A_{i,0}, \quad \mathbb {K}_i \mapsto \mathbb {K}_i, \quad \mathbb {K}_\delta \mapsto \mathfrak {C}, \end{aligned}$$

for $$i \in \mathbb {I}_0$$.

### The QSP braid group action

By [34, Lemma 2.7], for each $i \in \mathbb {I}$, there exists an automorphism $\textbf{T}_i$ of $\widetilde{\textbf{U}}^\imath$ such that $\textbf{T}_i(\mathbb {K}_\mu ) = \mathbb {K}_{s_i\mu }$ and

$$ \textbf{T}_i(B_j) = \left\{ \begin{array}{l l} \mathbb {K}_j^{-1}B_j &  \text{ if } i=j, \\[2pt] B_j &  \text{ if } a_{ji} = 0, \\[2pt] B_jB_i - q_iB_iB_j &  \text{ if } a_{ji} = -1, \\[2pt] {[2]_{q_i}^{-1}}(B_jB_i^2-q_i[2]_{q_i}B_iB_jB_i+q_i^2B_i^2B_j) + B_j\mathbb {K}_i &  \text{ if } a_{ji} = -2, \\[2pt] {[3]}_{q_i}^{-1}[2]_{q_i}^{-1}\big ( B_jB_i^3 - q_i[3]_{q_i}B_iB_jB_i^2 +q^2[3]_{q_i}B_i^2B_jB_i \\[2pt] -q_i^3B_i^3B_j + q_i^{-1}[B_j,B_i]_{q_i^3}\mathbb {K}_i\big ) + [B_j,B_i]_{q_i}\mathbb {K}_i &  \text{ if } a_{ji} = -3, \end{array} \right. $$

for $\mu \in {\mathbb Z}\mathbb {I}$ and $j \in \mathbb {I}$. For each $\lambda \in \Lambda$, there is also an automorphism $\textbf{T}_\lambda$ such that $\textbf{T}_\lambda (B_i) = B_{\lambda (i)}$ and $\textbf{T}_\lambda (\mathbb {K}_i) = \mathbb {K}_{\lambda (i)}$. These automorphisms define an action of the braid group $\widetilde{\mathcal {B}}$. We will refer to this action as the QSP braid group action to distinguish it from Lusztig’s braid group action from Sect. 2.2.

We will need the following lemma.

#### Lemma 2.4

We have $T_w(e^{\pm }_i) = e^{\pm }_{wi}$, $\textbf{T}_w(B_i) = B_{wi}$, for $i \in \mathbb {I}$ and $w \in {\widetilde{W}}$ such that $wi \in \mathbb {I}$.

#### Proof

See [28] and [34, Lemma 2.10]. $\square$

### Coideal structures

By [22, Theorem 7.1], for any $\textbf{s} = (s_0, \cdots , s_n) \in {\mathbb C}^{n+1}$, there exists an injective algebra homomorphism

2.4 $$\begin{aligned} \eta _{\textbf{s}}:\widetilde{\textbf{U}}^\imath \hookrightarrow \widetilde{\textbf{U}}, \quad B_i \mapsto e_i^- - q_i^{-2}\mathbb {K}_i e_i^+ K_i^{-1} + s_i K_i^{-1}, \quad \mathbb {K}_i \mapsto \mathbb {K}_i \quad (i \in \mathbb {I}). \end{aligned}$$

Given $\textbf{c} = (c_0, \cdots , c_n) \in ({\mathbb C}^\times )^{n+1}$, let $\textbf{U}^\imath _{\textbf{c}}$ be the quotient of $\widetilde{\textbf{U}}^\imath$ by the two-sided ideal generated by $q_i^{-2}\mathbb {K}_i - c_i$. The induced map $\eta _{\textbf{c}, \textbf{s}}:\textbf{U}^\imath _{\textbf{c}}\hookrightarrow \textbf{U}$ gives $\textbf{U}^\imath _{\textbf{c}}$ the structure of a right coideal subalgebra of $\textbf{U}$, with coproduct $\Delta _{\textbf{c},\textbf{s}} = \Delta \circ \eta _{\textbf{c},\textbf{s}}$. If $\textbf{s} = (0, \cdots , 0)$, we abbreviate $\eta = \eta _{\textbf{s}}$ and $\eta _{\textbf{c}} = \eta _{\textbf{c}, \textbf{s}}$. Explicitly,

2.5 $$\begin{aligned} \Delta _{\textbf{c},\textbf{s}}(B_i) = 1 \otimes \eta _{\textbf{c}}(B_i) + \eta _{\textbf{c}, \textbf{s}}(B_i) \otimes K_i^{-1}. \end{aligned}$$

### Rank one subalgebras

Let us recall how the braid group actions can be used to obtain rank one subalgebras in $\widetilde{\textbf{U}}$ and $\widetilde{\textbf{U}}^\imath$. For $i \in \mathbb {I}_0$, let $\omega ^{\prime }_i = \omega _i s_i$, and let $\textbf{U}_{[i]}$ be the subalgebra of $\textbf{U}$ generated by

$$ E_i, \ F_i, \ K_i^{\pm 1}, \ T_{\omega ^{\prime }_i}(E_i), \ T_{\omega ^{\prime }_i}(F_i), \ T_{\omega ^{\prime }_i}(K_i^{\pm 1}). $$

By [4, Proposition 3.8], there is an algebra isomorphism $\iota _i :U_q(L\mathfrak {sl}_2)\rightarrow \textbf{U}_{[i]}$ sending $q \mapsto q_i$ and

$$\begin{aligned}&E_1 \mapsto E_i, \ F_1 \mapsto F_i, \ K_1^{\pm 1} \mapsto K_i^{\pm 1}, \\&E_0 \mapsto T_{\omega ^{\prime }_i}(E_i), \ F_0 \mapsto T_{\omega ^{\prime }_i}(F_i), \ K_0^{\pm 1} \mapsto T_{\omega ^{\prime }_i}(K_i^{\pm 1}). \end{aligned}$$

Let $\widetilde{\textbf{U}}_{[i]}$ be the subalgebra of $\widetilde{\textbf{U}}$ generated by $\textbf{U}_{[i]}$, $\mathbb {K}_i^{\pm 1}$ and $(\mathbb {K}_\delta \mathbb {K}_i^{-1})^{\pm 1}$. The algebra isomorphism $\iota _i$ extends to an isomorphism $\iota _i :{\widetilde{U}}_q(L\mathfrak {sl}_2)\rightarrow \widetilde{\textbf{U}}_{[i]}$ sending $\mathbb {K}_1 \mapsto \mathbb {K}_i$ and $\mathbb {K}_0 \mapsto \mathbb {K}_\delta \mathbb {K}_i^{-1}$.

Moreover, for $i \in \mathbb {I}_0$, let $\widetilde{\textbf{U}}^\imath _{[i]}$ be the subalgebra of $\widetilde{\textbf{U}}^\imath$ generated by $B_i$, $\textbf{T}_{\omega ^{\prime }_i}(B_i)$, $\mathbb {K}_i^{\pm 1}$ and $(\mathbb {K}_\delta \mathbb {K}_i^{-1})^{\pm 1}$. By [29, Proposition 3.9], there is an algebra isomorphism $\iota :\widetilde{\textbf{U}}^\imath (\widehat{\mathfrak {sl}}_2) \rightarrow \widetilde{\textbf{U}}^\imath _{[i]}$ sending $q \mapsto q_i$ and

$$\begin{aligned} B_1 \mapsto B_i, \quad B_0 \mapsto \textbf{T}_{\omega ^{\prime }_i}(B_i), \quad \mathbb {K}_1 \mapsto \mathbb {K}_i, \quad \mathbb {K}_0 \mapsto \mathbb {K}_\delta \mathbb {K}_i^{-1}. \end{aligned}$$

We also note that $A_{1,-1}$ is sent to $\textbf{T}_{\omega _i}(B_i)$.

### Factorization and coproduct in rank one

Let $\mathcal {H}$ denote the commutative subalgebra of $\widetilde{\textbf{U}}$ generated by the coefficients $\phi ^{\pm }_{i,\pm r}$ of the series $\pmb {\phi }^{\pm }_i(z)$. We will refer to these coefficients as the Drinfeld–Cartan operators and to $\mathcal {H}$ as the Drinfeld–Cartan subalgebra of $\widetilde{\textbf{U}}$. Similarly, we refer to the coefficients of the series

$$ \pmb {\grave{\Theta }}_i(z) = (q_i-q_i^{-1})\frac{1-q_i^{-2}\mathfrak {C}z^2}{1-\mathfrak {C}z^2} \sum _{r \ge 0} \Theta _{i,r} z^r $$

as $\imath$Drinfeld–Cartan operators^2^, and to the commutative subalgebra $\mathcal {H}^\imath$ generated by them as the $\imath$Drinfeld–Cartan subalgebra of $\widetilde{\textbf{U}}^\imath$. It is natural to ask how the two Drinfeld–Cartan subalgebras are related under the monomorphism (2.4). This question was answered in [32] for quantum symmetric pairs of split affine type $\textsf{A}$. Here, we will need the rank one case of that result, i.e., when $\widetilde{\textbf{U}}^\imath$ is isomorphic to the (universal) *q*-Onsager algebra.

#### Theorem 2.5

[ [32, Theorem 7.5]] Let $\widehat{{\mathfrak {g}}} = \widehat{\mathfrak {sl}}_2$. The series $\pmb {\grave{\Theta }}(z)$ admits the following factorization

$$\begin{aligned} \eta _{\textbf{s}}(\pmb {\grave{\Theta }}(z)) \equiv&\ \xi _{\textbf{s}}(\pmb {\grave{\Theta }}(z)) \cdot \pmb {\phi }^-(z^{-1})\pmb {\phi }^+(\mathfrak {C}z) \quad \mod {\widetilde{\textbf{U}}_+}[\negthinspace [z]\negthinspace ], \\ \Delta _{\textbf{s}}(\pmb {\grave{\Theta }}(z)) \equiv&\ \eta _{\textbf{s}}(\pmb {\grave{\Theta }}(z)) \otimes \eta (\pmb {\grave{\Theta }}(z)) \quad \mod {\widetilde{\textbf{U}}\otimes \widetilde{\textbf{U}}_{+}}[\negthinspace [z]\negthinspace ], \end{aligned}$$

where $$\xi _{\textbf{s}}= \varepsilon \circ \eta _{\textbf{s}}$$.

## Reduced expressions for fundamental weights

The goal of this section is to obtain explicit reduced expressions for the extended affine Weyl group elements corresponding to the fundamental weights in types $\textsf{A}_n^{(1)}, \textsf{B}_n^{(1)}$, $\textsf{C}_n^{(1)}, \textsf{D}_n^{(1)}$ (Table 2).

First, we need to introduce some notations. Let $w_0$ be the longest element of the finite Weyl group $W_0$ of ${\mathfrak {g}}$. Given $i \in \mathbb {I}_0$, let $w_i$ be the longest element of the Weyl group corresponding to the Dynkin diagram of ${\mathfrak {g}}$ with the node labeled by *i* removed. Given $1 \le k < l$, let

$$\begin{aligned} [k,l] = s_k s_{k+1} \cdots s_{l}, \quad [l,k] = [k,l]^{-1}. \end{aligned}$$

Given the highest root $\theta = \sum _{i \in \mathbb {I}_0} c_i \alpha _i$, let $J = \{ i \in \mathbb {I}_0 \mid c_i = 1\}$.

We will use the following two results.

### Proposition 3.1

[ [7, Ch. VI §2.3, Proposition 6], [20, Proposition 1.18]] There is a bijection $J \longrightarrow \Lambda {-} \{1\}$ sending $i \mapsto \omega _i w_i w_0$.

### Proposition 3.2

[ [20, Proposition 1.23]] The length of the fundamental weights is given by

$$ \ell (\omega _i) = \sum _{\beta \in \Delta ^+}(\beta , \omega _i), $$

where $\Delta ^+$ is the set of the positive roots of ${\mathfrak {g}}$.


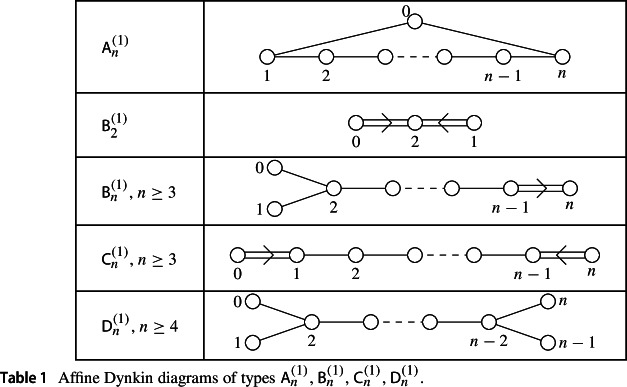


### Type $\textsf{A}_n^{(1)}$

The highest root is $\theta = \alpha _1 + \alpha _2 + \cdots + \alpha _n$ and $J = \mathbb {I}_0$. The fundamental group $\Lambda$ is cyclic of order $n+1$. It is generated by, e.g., the automorphism $\pi$ of the affine Dynkin diagram sending $i \mapsto i+1 \mod n+1$.

#### Proposition 3.3

Let ${\widetilde{W}}$ be the extended affine Weyl group of type $A_n^{(1)}$, for $n \ge 1$. Then,

$$ \omega _i = \pi ^i [n - i+1 ,n] \cdots [2,i+1][1,i]. $$

This is a reduced expression.

#### Proof

See [27, §4.5] or [32, Proposition 9.2]. $\square$

### Type $\textsf{D}_n^{(1)}$

The highest root is $\theta = \alpha _1 + \alpha _{n-1} + \alpha _n + 2(\alpha _2 + \cdots + \alpha _{n-2})$ and $J = \{1, n{-}1, n\}$. The fundamental group $\Lambda = \{ 1, \pi _1, \pi _{n-1}, \pi _n \}$ depends on the parity of *n*. If *n* is even then $\Lambda \cong {\mathbb Z}/2{\mathbb Z}\times {\mathbb Z}/2{\mathbb Z}$, and if *n* is odd, then $\Lambda \cong {\mathbb Z}/4{\mathbb Z}$. Explicit descriptions of the corresponding diagram automorphisms can be found in [7, Ch. VI §4.8]. Given $1 \le m \le n-2$, set

#### Proposition 3.4

Let ${\widetilde{W}}$ be the extended affine Weyl group of type $\textsf{D}_n^{(1)}$, for $n \ge 4$. If *i* is even, thenIf *i* is odd, thenMoreover, the formulae above yield reduced expressions.

#### Proof

If $i \in J = \{1,n{-}1,n\}$, one may use Proposition 3.1 and argue in a similar way as in Proposition 3.3. For more general $i \in \mathbb {I}_0$, consider ${\widetilde{W}}$ as the group of affine transformations of ${\mathbb R}^n$. We show that elements on the RHS of the equations above act in the same way as $\omega _i$. Indeed, the element  acts as a cyclic permutation of the coordinates (shifting the indices by $n-i$). It is also easy to explicitly verify that  (in the even case) and  (in the odd case) act as cyclic permutations of the coordinates (shifting the indices by *i*) followed by an affine shift by $\omega _i$.

We will prove the reducedness of our expressions for $\omega _i$ by induction on *n*. The base case of $\textsf{D}_4$ can be checked directly. Now, assume that $n \ge 5$, and that the result is true for $\textsf{D}_{n-1}$. The set of positive roots of type $\textsf{D}_{n}$ ($n \ge 5$) is the union of the set of positive roots of type $\textsf{D}_{n-1}$ (with the indices shifted by 1), and the set consisting of the roots

3.1 $$\begin{aligned} \sum _{j=1}^l \alpha _j \ (1 \le l \le n), \quad \alpha _n+\sum _{j=1}^{n-2}\alpha _j, \quad \sum _{j=1}^{n} \alpha _j + \sum _{j=2}^{l} \alpha _{n-j} \ (1 \le l \le n-2). \end{aligned}$$

Let $$1 \le i \le n-2$$. We will show that $$\ell (\omega _i) = i(2n-i-1)$$. By Proposition 3.2, it suffices to check that the sum of the coefficients of $$\alpha _i$$ in positive roots of type $$\textsf{D}_n$$ (written in terms of the basis of simple roots) is equal to $$i(2n-i-1)$$. This sum is equal to the sum of the coefficients of $$\alpha _{i-1}$$ in the positive roots of type $$\textsf{D}_{n-1}$$ and the sum of the coefficients of $$\alpha _i$$ in the roots in (3.1). By induction, the former is equal to $$(i-1)(2n-2-i)$$. Therefore, the total sum is $$ (i-1)(2n-2-i) + (n-i+1) + 1 + (n-3) + (i-1) = i(2n-i-1)$$.

Next, let $i=n$. We will check that $\ell (\omega _n)=n(n-1)/2$. By induction, the sum of the coefficients of $\alpha _{n-1}$ in the positive roots of type $\textsf{D}_{n-1}$ is $(n-1)(n-2)/2$. Therefore, the total sum of the coefficients of $\alpha _{n}$ in the positive roots of type $\textsf{D}_{n}$ is $(n-1)(n-2)/2+n-1 = n(n-1)/2$.

Finally, let $i=n-1$. We will check that $\ell (\omega _n)=n(n-1)/2$. By induction, the sum of the coefficients of $\alpha _{n-2}$ in the positive roots of type $\textsf{D}_{n-1}$ is $(n-1)(n-2)/2$. Therefore, the total sum of the coefficients of $\alpha _{n-1}$ in the positive roots of type $\textsf{D}_{n}$ is $(n-1)(n-2)/2+n-1 = n(n-1)/2$.

In each of the three cases, the length of $\omega _i$ equals the number of letters in the expressions for $\omega _i$ we found, implying that these expressions are reduced. $\square$

### Type $\textsf{B}_n^{(1)}$

The highest root is $\theta =\alpha _1+ 2\sum _{i=2}^n \alpha _i$ and $J=\{1\}$. The fundamental group is $\Lambda = \{ 1, \pi _1\}$, where $\pi _1$ interchanges $\alpha _0$ and $\alpha _1$ and fixes the other $\alpha _j^{\prime }$s.

#### Proposition 3.5

Let ${\widetilde{W}}$ be the extended affine Weyl group of type $\textsf{B}_n^{(1)}$, for $n \ge 2$. If *i* is odd, then^3^If *i* is even, then^4^Moreover, the formulae above yield reduced expressions.

#### Proof

Given $I \subset \{1, n\}$, let $w_0^I$ denote the longest word in the parabolic subgroup $W_I$.

First consider the case of $i=1$. By Proposition 3.1, $\omega _1 = \pi _1 w_0 w_1$. We have $w_0 = w_0^{[1,n-1]} (s_n (s_{n-1}s_n) \cdots [2,n])^{-1}$ and $w_1 = s_n (s_{n-1}s_n) \cdots [2,n] w_0^{[2,n-1]}$. Therefore $\pi _1 w_0 w_1 = \pi _1 w_0^{[1,n-1]} w_0^{[2,n-1]} = \pi _1 [1,n] [n-1,1]$.

For $i \ne 1$, we use the same strategy as in the proof of Proposition 3.4, i.e., show that the expressions we found define the same affine transformations of ${\mathbb R}^n$. More precisely, we need to show that each $\omega _i$ (as an element of the extended affine Weyl group of type $\textsf{B}_n$) acts as a translation by the *i*-th fundamental weight of type $\textsf{C}_n$. Let us first recall some facts about the root systems of types $\textsf{B}_n$ and $\textsf{C}_n$. The simple roots in the root system of type $\textsf{B}_n$ are

$$\begin{aligned} \alpha _j = e_j - e_{j+1}\ (1 \le j \le n-1), \ \ \alpha _n=e_n. \end{aligned}$$

We have $$\theta =\alpha _1+2\sum _{i=2}^n \alpha _i = e_1+e_2=\theta ^{\vee }$$,

$$\begin{aligned} s_j(e_j) =&\ e_{j+1} \    &   (1 \le j \le n-1), \quad&s_n(e_n)&= \ -e_n, \\ s_j(e_{j+1}) =&\ e_j \    &   (1 \le j \le n-1), \quad&s_0(e_j)&= \ t_{\theta ^\vee } r_\theta (e_j), \end{aligned}$$

where $t_{\theta ^\vee }$ is the translation by ${\theta ^\vee }$; $r_\theta$ is the reflection by $\theta$; and in all other cases $s_j(e_{j^{\prime }}) = e_{j^{\prime }}$. Finally, recall that the fundamental weights of type $\textsf{C}_n$ are $\omega _i^* = \sum _{j=1}^i e_j$ ($1 \le i \le n$).

Observe that $[n-i,n-1] \cdots [2,i+1] [1,i]$ acts by cyclically shifting the coordinates by $n-i$. Let *i* be even. We have

$$\begin{aligned}&\left( s_0[2,n][1,n]\right) ^{\frac{i}{2}} [n-i, n-1] \cdots [2,i+1] [1,i] (e_j) = \left( s_0[2,n][1,n]\right) ^{\frac{i}{2}} \left( e_{n-i+j}\right) \\&\quad = \left( s_0[2,n][1,n]\right) ^{\frac{i}{2}-1} t_{\theta } \left( e_{j-i+2}\right) = \left( s_0[2,n][1,n]\right) ^{\frac{i}{2}-1} \left( e_{j-i+2} + e_1 + e_2\right) \\&\quad = \left( s_0[2,n][1,n]\right) ^{\frac{i}{2}-2} \left( e_{j-i+4} + e_1+e_2 + e_3 + e_4\right) = e_j + \sum _{r=1}^i e_r = e_j + \omega _i^*. \end{aligned}$$

Let *i* be odd. We have

$$\begin{aligned}&\pi _1 \left( [1,n]s_0[2,n]\right) ^{\frac{i-1}{2}}[1,n] [n-i,n-1] \cdots [2,i+1][1,i] (e_j) \\&\qquad = \pi _1 \left( [1,n]s_0[2,n]\right) ^{\frac{i-1}{2}} [1,n] (e_{n-i+j}) = \pi _1 [1,n] (s_0[2,n][1,n])^{\frac{i-1}{2}} (e_{n-i+j}) \\&\qquad = \pi _1 [1,n] (s_0[2,n][1,n])^{\frac{i-3}{2}} \left( e_{j-i+2} + e_1+e_2\right) = \pi _1 [1,n] \left( e_{j-1} + \sum _{r=1}^{i-1} e_r\right) \\&\qquad = t_{\omega _1^*} [1,n-1] (e_{j-1} + \sum _{r=1}^{i-1} e_r) = t_{\omega _1^*} \left( e_{j} + \sum _{r=2}^i e_r \right) = e_{j} + \omega _i^*, \end{aligned}$$

where in the passage from the third to the fourth lines we used the already established formula for $\omega _1$.

We will now prove, by induction on *n*, that the expressions for $\omega _i$ we produced are reduced. The base case $n=2$ can be checked directly. So assume that $n \ge 3$ and the result is true for $\textsf{B}_{n-1}$. The set of positive roots of type $\textsf{B}_{n}$ ($n \ge 3$) is the union of the set of positive roots of type $\textsf{B}_{n-1}$ (with indices shifted by 1) and the set consisting of the roots

3.2 $$\begin{aligned} \sum _{j=1}^l \alpha _j \ (1 \le l \le n), \quad \sum _{j=1}^{n} \alpha _j + \sum _{j=1}^{l} \alpha _{n-j+1} \ (1 \le l \le n-1). \end{aligned}$$

Let $$1 \le i \le n$$. We will check that $$\ell (\omega _i) = i(2n-i)$$. By Proposition 3.2, it suffices to check that the sum of the coefficients of $$\alpha _i$$ in positive roots of type $$\textsf{B}_n$$ is equal to $$i(2n-i)$$. This sum is equal to the sum of the coefficients of $$\alpha _{i-1}$$ in positive roots of type $$\textsf{B}_{n-1}$$ and the sum of the coefficients of $$\alpha _i$$ in the positive roots in (3.2). By induction, the former is equal to $$(i-1)(2n-2-i+1)$$. Therefore, the total sum is $$ (i-1)(2n-2-i+1) + (n-i+1) + (n-1) + (i-1) = i(2n-i)$$. This is also the length of the expressions for $$\omega _i$$ we found, implying that they are indeed reduced.

### Type $\textsf{C}_n^{(1)}$

The highest root is $\theta =\alpha _n + 2\sum _{i=1}^{n-1} \alpha _i$ and $J=\{n\}$. The fundamental group is $\Lambda = \{ 1, \pi _n\}$, where $\pi _n$ interchanges $\alpha _k$ and $\alpha _{n-k}$.

#### Proposition 3.6

Let ${\widetilde{W}}$ be the extended affine Weyl group of type $\textsf{C}_n^{(1)}$, for $n \ge 2$. If $i=n$, then

3.3 $$\begin{aligned} \omega _n = \pi _n s_n[n-1,n] \cdots [1,n]. \end{aligned}$$

If $$i<n$$, then

$$\begin{aligned} \omega _i = (s_0 [1,n] )^i [n-i,n-1] \cdots [2,i+1][1,i]. \end{aligned}$$

Moreover, the formulae above yield reduced expressions.

#### Proof

We follow the same strategy as in the proofs for types $\textsf{B}_n^{(1)}$ and $\textsf{D}_n^{(1)}$. The simple roots in the root system of type $\textsf{C}_n$ are

$$\begin{aligned} \alpha _j = e_j - e_{j+1} \ (1 \le j \le n-1), \ \ \alpha _n = 2 e_n. \end{aligned}$$

The highest root is $\theta = 2 e_1 = e_n + 2 \sum _{i=1}^{n-1} e_i$ and $\theta ^{\vee } = e_1$. We have

$$\begin{aligned} s_j(e_j) =&\ e_{j+1} \    &   (1 \le j \le n-1), \quad&s_n(e_n) =&\ -e_n, \\ s_j(e_{j+1}) =&\ e_j \    &   (1 \le j \le n-1), \quad&s_0(e_j) =&\ t_{\theta ^\vee } r_\theta (e_j), \end{aligned}$$

and in all other cases $s_j(e_{j^{\prime }}) = e_{j^{\prime }}$. Moreover, the fundamental weights of the dual root system $\textsf{B}_n$ are $\omega _i^* = \sum _{j=1}^i e_j$ ($i<n$), and $\omega _n^* =\frac{1}{2} \sum _{j=1}^n e_j$.

The case of $i=n$ can simply be handled using Proposition 3.1, i.e., $\omega _n = \pi _n w_0 w_n = \pi _n s_n(s_{n-1}s_n) \cdots [1,n]$. Next, suppose that $i<n$. We have

$$\begin{aligned}&(s_0 [1,n])^i [n-i,n-1] \cdots [2,i+1][1,i] (e_j) = (s_0 [1,n])^i (e_{n-i+j}). \end{aligned}$$

If $$j=i$$, then

$$\begin{aligned} (s_0 [1,n])^i (e_{n})&= (s_0 [1,n])^{i-1} t_{\theta ^{\vee }}r_{\theta } (-e_1) = (s_0 [1,n])^{i-1} (2e_1) \\&= (s_0 [1,n])^{i-2} (e_1+2e_2) = (s_0 [1,n])^{i-3} (e_1+e_2+2e_3)\\  &= e_i + \sum _{r=1}^i e_r = \omega _i^* + e_i. \end{aligned}$$

If $i<j$, then $j-i+1 \ge 2$ and

$$\begin{aligned}&(s_0 [1,n])^i (e_{n-i+j}) = (s_0 [1,n])^i (e_{j-i}) = (s_0 [1,n])^{i-1} t_{\theta ^{\vee }} r_{\theta } (e_{j-i+1}) \\&\quad = (s_0 [1,n])^{i-1} (e_1+e_{j-i+1}) = (s_0 [1,n])^{i-2} (e_1+e_2+e_{j-i+2})\\&\quad = e_j + \sum _{r=1}^i = e_j + \omega _i^*. \end{aligned}$$

If $$j<i$$, then

$$\begin{aligned}&(s_0 [1,n] )^i (e_{n-i+j})\\&\quad = (s_0 [1,n] )^{i-1} (e_1+e_{n-i+j+1}) = (s_0 [1,n] )^{i-2} (e_1+e_2+e_{n-i+j+2}) \\&\quad = (s_0 [1,n] )^{j} ( e_n + \sum _{r=1}^{i-j} e_r ) = (s_0 [1,n] )^{j-1} (e_1 + \sum _{r=1}^{i-j+1} e_r )\\&\quad =e_j+ \sum _{r=1}^{i} e_r =e_j + \omega _i^*. \end{aligned}$$

We will now prove, by induction on *n*, that the expressions for $\omega _i$ we found are reduced. The base case of $\textsf{C}_2$ can be checked directly. So assume that $n \ge 3$ and the result is true for $\textsf{C}_{n-1}$. The set of positive roots of type $\textsf{C}_{n}$ ($n \ge 3$) is the union of the set of positive roots of type $\textsf{C}_{n-1}$ (with the indices shifted by 1) and the set consisting of the roots

$$\begin{aligned} \sum _{j=1}^l \alpha _j \ (1 \le l \le n), \quad \sum _{j=1}^{n} \alpha _j + \sum _{j=1}^{l} \alpha _{n-j} \ (1 \le l \le n-1). \end{aligned}$$

We will check that $\ell (\omega _i) = i(n+1)$ if $i<n$ and $\ell (\omega _n)=n(n+1)/2$. By Proposition 3.2, it suffices to check that the sum of coefficients of $\alpha _i$ in positive roots of type $\textsf{C}_n$ is equal to $i(n-1)$ if $i<n$, and to $n(n+1)/2$ if $i=n$. This sum is equal to the sum of the coefficients of $\alpha _{i-1}$ in positive roots of type $\textsf{C}_{n-1}$ and the sum of the coefficients of $\alpha _i$ in the positive roots in (3.4).

Let $$i=n$$. By induction hypothesis, the sum of the coefficients of $$\alpha _{n-1}$$ in positive roots of type $$\textsf{C}_{n-1}$$ is equal to $$(n-1)n/2$$. Therefore, the total sum is $$ (n-1)n/2 + n = n(n+1)/2$$. Now, let $$i<n$$. By induction hypothesis, the sum of the coefficients of $$\alpha _{i-1}$$ in positive roots of type $$\textsf{C}_{n-1}$$ is equal to $$(i-1)n$$. Therefore, the total sum is $$ (i-1)n + (n-i+1) + (n-1) + i = i(n+1)$$, as claimed. This is also the length of the expressions for $$\omega _i$$ we found, implying that they are indeed reduced.

## Auxiliary calculations—root combinatorics

We will need the following lemmas to compute the braid group action in Sect. 7, with the aid of Lemma 2.4. Their proofs are based on straightforward calculations, so we omit most of the details.

### Type $\textsf{D}_n^{(1)}$

We consider the cases of weights $\omega _1, \cdots , \omega _{n-2}$ and $\omega _{n-1}, \omega _n$ separately.

#### Weights $\omega _1, \cdots , \omega _{n-2}$

##### Lemma 4.1

We have

##### Proof

The lemma follows by direct calculation. $\square$

Set

$$\begin{aligned} {\tilde{\alpha }}_k =&\ \left\{ \begin{array}{r l} \alpha _k &  \quad \text{ if } \ 1 \le k \le n-1, \\ \alpha _0 + \alpha _2 + \cdots + \alpha _{n-2} + \alpha _n &  \quad \text{ if } \ k = 0. \end{array} \right. \\ {\hat{\alpha }}_k =&\ \left\{ \begin{array}{r l} \alpha _k &  \quad \text{ if } \ 1 \le k \le n-2, \\ \alpha _{n} &  \quad \text{ if } \ k = n-1, \\ \alpha _0 + \alpha _2 + \cdots + \alpha _{n-2} + \alpha _{n-1} &  \quad \text{ if } \ k = 0. \end{array} \right. \end{aligned}$$

For $$1 \le i \le i-2$$, set

##### Lemma 4.2

For $1 \le k \le n-1$, we have

$$ \zeta _i \cdot \alpha _k = \left\{ \begin{array}{r l} {\tilde{\alpha }}_{k+i} &  \quad \text{ if } \ i \ \text{ is } \text{ even }, \\ {\hat{\alpha }}_{k+i} &  \quad \text{ if } \ i \ \text{ is } \text{ odd }, \end{array} \right. $$

##### Proof

Using Lemma 4.1, we observe that the set $\{ {\tilde{\alpha }}_k\}_{1 \le k \le n}$ is closed under the action of , and that this action sends ${\tilde{\alpha }}_k \mapsto {\tilde{\alpha }}_{k+1}$ (with indices modulo *n*). Repeating this action $\frac{i}{2}$-times, when *i* is even, yields the first case of the lemma.

Moreover, it follows that if *i* is odd, then  acts by sending ${\tilde{\alpha }}_k \mapsto {\tilde{\alpha }}_{k+i-1}$. Using Lemma 4.1 again, we observe that  defines a bijection

$$ \{ {\tilde{\alpha }}_k\}_{1 \le k \le n} \rightarrow \{ {\hat{\alpha }}_k\}_{1 \le k \le n}, \qquad {\tilde{\alpha }}_k \mapsto {\hat{\alpha }}_{k+1}. $$

$$\square $$

#### Weights $\omega _{n-1}, \omega _n$

Set

##### Lemma 4.3

We have

##### Proof

The lemma follows by a direct calculation. $\square$

### Type $\textsf{B}_n^{(1)}$

For $$1 \le i \le n$$, set

4.1 $$\begin{aligned} \zeta _i = {\left\{ \begin{array}{ll} \left( s_0[2,n][1,n]\right) ^{\frac{i}{2}}, &  \text {if }i\text { is even}, \\ \pi _1 [1,n]\left( s_0[2,n][1,n]\right) ^{\frac{i-1}{2}} &  \text {if }i\text { is odd}. \end{array}\right. } \end{aligned}$$

Set

$$\begin{aligned} {\tilde{\alpha }}_k = {\left\{ \begin{array}{ll} \alpha _k &  1 \le k \le n-1, \\ \alpha _0 + 2\alpha _n + \sum _{j=2}^{n-1} \alpha _j &  k=0. \end{array}\right. } \end{aligned}$$

#### Lemma 4.4

If $1 \le k \le n-1$, then $\zeta _i.\alpha _k = {\tilde{\alpha }}_{i+k}$, where the indices are taken modulo *n*.

#### Proof

The proof is similar to that of Lemma 4.3. $\square$

### Type $\textsf{C}_n^{(1)}$

Let $\zeta _i = (s_0s_1\cdots s_n)^i$, for $1 \le i \le n-1$, and set

$$\begin{aligned} {\tilde{\alpha }}_k = {\left\{ \begin{array}{ll} \alpha _k &  1 \le k \le n-1, \\ \sum _{j=0}^{n} \alpha _j &  k=0. \end{array}\right. } \end{aligned}$$

#### Lemma 4.5

For $1 \le i,k \le n-1$, we have $\zeta _i.\alpha _k = {\tilde{\alpha }}_{i+k}$, where the indices are taken modulo *n*.

#### Proof

The lemma follows by a direct calculation. $\square$

## Good polynomials

The goal of this section is to formulate explicit criteria which will allow us to deduce the compatibility of the new Drinfeld presentations for $\widetilde{\textbf{U}}$ and $\widetilde{\textbf{U}}^\imath$. From now on, assume that $\textbf{s} = (0, \cdots , 0)$ and abbreviate ${\widetilde{E}}_i = - q_i^{-2} \mathbb {K}_i E_i K_i^{-1}$.

### Iterated *q*-brackets

As shown in Sect. 3, reduced expressions for the fundamental weights in all classical types contain subexpressions almost identical to the formulae encountered in type $\textsf{A}$. Below we describe the braid group action corresponding to such subexpressions in terms of iterated *q*-brackets.

Define non-commutative polynomials $P_k(y_1, \cdots , y_k)$ and $P^{\prime }_k(y_1, \cdots , y_k)$ over ${\mathbb C}$ by induction in the following way:

$$\begin{aligned} P_1(y_1) =&\ y_1, \qquad&P_{k+1}(y_1, \cdots , y_{k+1}) =&\ P_k(y_1, \cdots , y_{k-1}, [y_k,y_{k+1}]_q), \\ P^{\prime }_1(y_1) =&\ y_1, \qquad&P^{\prime }_{k+1}(y_1, \cdots , y_{k+1}) =&\ [P^{\prime }_k(y_1, \cdots , y_k), y_{k+1}]_q. \end{aligned}$$

Clearly, we have

$$\begin{aligned} P_k(y_1, \cdots , y_k) =&\ P_{l+1}(y_1, \cdots , y_l, P_{k-l}(y_{l+1}, \cdots , y_k)), \\ P^{\prime }_k(y_1, \cdots , y_k) =&\ P^{\prime }_{k-l+1}(P^{\prime }_l(y_1, \cdots , y_l), y_{l+1}, \cdots , y_k). \end{aligned}$$

for any $$1 \le l \le k-1$$.

We say that a tuple $(y_1, \cdots \!, y_k)$ is *almost commuting* if $y_my_n \!=\! y_ny_m$, for $|m-n| \!>\! 1$.

#### Lemma 5.1

The polynomials $P_k$ and $P^{\prime }_k$ are equal if $(y_1, \cdots , y_k)$ is almost commuting. In particular, in that case,

$$ P_2\left( y_1, P_2(y_2, y_3)\right) = P^{\prime }_2\left( P^{\prime }_2(y_1, y_2), y_3\right) . $$

#### Proof

The first statement is proven by induction. By definition, $P_1 = P^{\prime }_1$ and $P_2 = P^{\prime }_2$. Moreover,

$$\begin{aligned} P_{k+1}\left( y_1, \cdots , y_{k+1}\right) =&\ P_2\left( y_1, P_k(y_2, \cdots ,y_{k+1})\right) \\ =&\ P_2\left( y_1, P^{\prime }_k(y_2, \cdots ,y_{k+1})\right) \\ =&\ P^{\prime }_k\left( P_2(y_1, y_2), y_3, \cdots , y_{k+1}\right) \\ =&\ P^{\prime }_k\left( P^{\prime }_2(y_1, y_2), y_3, \cdots , y_{k+1}\right) = P^{\prime }_{k+1}\left( y_1, \cdots , y_{k+1}\right) , \end{aligned}$$

where in the second equality we used induction, and in the third equality the fact that the tuple is almost commuting. For the second part, observe that

$$ P_2\left( y_1, P_2(y_2, y_3)\right) = P_3(y_1, y_2, y_3) = P^{\prime }_3(y_1, y_2, y_3) = P^{\prime }_2(P^{\prime }_2(y_1,y_2), y_3). $$

$$\square $$

#### Lemma 5.2

If $$y_my_{m+1} = y_{m+1}y_m$$, then

$$ P_k(\cdots , y_m, y_{m+1}, \cdots ) = P_k(\cdots , y_{m+1}, y_{m}, \cdots ). $$

#### Proof

It suffices to consider the case $k=3$ with $[y_1, y_2] = 0$. Then,

$$\begin{aligned} P_3(y_1, y_2, y_3) =&\ y_1y_2y_3 - q(y_1y_3y_2 + y_2y_3y_1) + q^2 y_3 y_2 y_1 \\ =&\ y_2y_1y_3 - q(y_1y_3y_2 + y_2y_3y_1) + q^2 y_3 y_1 y_2 = P_3(y_2, y_1, y_3). \end{aligned}$$

$$\square $$

We say that an ordered tuple $(i_1, \cdots , i_k)$ of mutually distinct elements of $\mathbb {I}$ is a *chain* if $a_{i_l,i_{l+1}} a_{i_{l+1},i_{l}} = 1$ for each . We can identify the Dynkin subdiagram formed by $i_1, \cdots , i_k$ with a Dynkin diagram of type $\textsf{A}_k$. To avoid double subscripts, we relabel $i_1 = 1$, etc. Let us abbreviate5.1for $1 \le l \le k$.

#### Lemma 5.3

Let $(1, \cdots , k)$ be a chain in $\mathbb {I}$.

1. We have
  $$ \textbf{T}_{[k,2]} (B_{1}) = P_k(B_{1}, \cdots , B_{k}). $$
  Similarly,
  $$ T_{[k,2]} (F_{1}) = P_k(F_{1}, \cdots , F_{k}), \qquad T_{[k,2]} ({\widetilde{E}}_{1}) = P_k({\widetilde{E}}_{1}, \cdots , {\widetilde{E}}_{k}). $$
2. For $1 \le l \le k$, we have
  $$ \textbf{T}_{\tau _{l}} (B_{l}) = P_l(B_{k}, \cdots , B_{k-l+2}, P_{k-l+1}(B_{1}, \cdots , B_{k-l+1})). $$
  The same formula holds if $\textbf{T}_{\tau _{l}}$ is replaced by $T_{\tau _{l}}$, and every $B_i$ uniformly replaced by $F_i$ or ${\widetilde{E}}_i$.

#### Proof

This is proven in [32, Lemmas 9.3-$-$9.4, Proposition 9.6]. $\square$

Later, we will also need the polynomials

5.2 $$\begin{aligned} \textbf{P}_i(a,b) = \sum _{r=0}^2 q^r b^{(2-r)} a b^{(r)} + \mathbb {K}_i a, \qquad \widehat{\textbf{P}}(a,b) = \sum _{r=0}^2 q^r b^{(2-r)} a b^{(r)}, \end{aligned}$$

for $$i \in \mathbb {I}$$.

### Good polynomials

Let $P(B_0, \cdots , B_n)$ be any non-commutative polynomial in the variables $B_0, \cdots , B_n$ with coefficients in ${\mathbb C}(\mathbb {K}_i)_{i \in \mathbb {I}}$. Considering this polynomial as an element of $\widetilde{\textbf{U}}$, and substituting for an occurrence of a letter $B_j$ either ${\widetilde{E}}_j$ or $F_j$, we can write it as a sum of polynomials which are homogeneous^5^ in each of the variables ${\widetilde{E}}_j$ and $F_j$:

$$\begin{aligned} \eta \left( P(B_0, \cdots , B_n)\right) = \sum _{\underline{d}} P_{\underline{d}}\left( {\widetilde{E}}_0, F_0, \cdots , {\widetilde{E}}_n, F_n\right) , \end{aligned}$$

where $\underline{d} = (d_j^\pm )_{j \in \mathbb {I}}$, with $d_j^+ = \deg _{{\widetilde{E}}_j}P_{\underline{d}}(\cdot )$ and $d_j^- = \deg _{F_j} P_{\underline{d}}(\cdot )$.^6^ We call the summands on the RHS *subterms*, and the corresponding tuple $\underline{d}$ the type of a subterm. We say that that a subterm is *mixed* if there exist $k,l \in \mathbb {I}$ such that $d_k^+, d_l^- > 0$; otherwise, a subterm is called *pure*.

We will now introduce some technical assumptions and definitions with view to proving Proposition 5.5. From this point on, let us assume that each subterm satisfies $d_0^+ + d_0^- = 1$. Let $P_+$ be the sum of all subterms with $d_0^+ = 1$, and $P_-$ be the sum of all subterms with $d_0^- = 1$. Moreover, let $P_{--}$ be the sum of all subterms which have maximal total degree and $d_j^+ = 0$ for all $j \in \mathbb {I}$.

#### Definition 5.4

Let $i \in \mathbb {I}_0$. A polynomial $P(B_0, \cdots , B_n)$ is called:

1. *good* if every mixed subterm of $P(B_0, \cdots , B_n)$ with $d_0^+ > 0$ vanishes;
2. *i*-*good* if it satisfies the following conditions:
  - every subterm $P_{\underline{d}}({\widetilde{E}}_0, F_0, \cdots , {\widetilde{E}}_n, F_n)$ satisfies^7^:
    5.3 $$\begin{aligned} \sum _{1 \le j \le n} (d_j^+ + d_j^-) \alpha _{j} \le \theta - \alpha _i, \qquad d_0^+ + d_0^- = 1, \end{aligned}$$
  - $$P_+ = T_{\omega ^{\prime }_i}({\widetilde{E}}_i)$$, $$P_{--} = T_{\omega ^{\prime }_i}(F_i)$$.

The importance of *i*-good polynomials is conveyed by the following proposition.

#### Proposition 5.5

Suppose that $\textbf{T}_{\omega ^{\prime }_i}(B_i)$ can be expressed as an *i*-good polynomial. Then,

5.4 $$\begin{aligned} \eta (\textbf{T}_{\omega _i^{\prime }}(B_i)) \equiv T_{\omega _i^{\prime }}(\eta (B_i)) \quad \mod \widetilde{\textbf{U}}_{d_i \ge 1,+}, \end{aligned}$$

and the diagram 
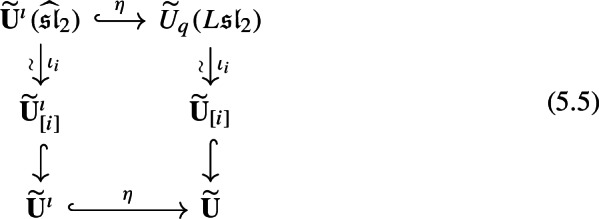
 commutes modulo $$\widetilde{\textbf{U}}_{\ne i,+}$$.

#### Proof

Suppose that $\textbf{T}_{\omega ^{\prime }_i}(B_i) = P(B_0, \cdots , B_n)$ is an *i*-good polynomial. Definition 5.4 implies that

5.6 $$\begin{aligned} \eta (P(B_0, \cdots , B_n)) = P_+ + P_- = T_{\omega ^{\prime }_i}({\widetilde{E}}_i) + P_-. \end{aligned}$$

Let us analyze $P_-$ further. It can be written as the sum of (i) $P_{--} = T_{\omega ^{\prime }_i}(F_i)$, (ii) pure subterms with $\sum _{1 \le j \le n} d_j^- \alpha _{j} < \theta - \alpha _i$, and (iii) mixed subterms, with $d_0^- = 1$ in each case. It follows from Lemma 2.1 and (5.3) that the degrees of these subterms (in the Drinfeld gradation) are given by

$$ (i) \ \alpha _i , \quad (ii) \ \theta - \sum _{1 \le j \le n} d_j^- \alpha _{j}> \alpha _i, \quad (iii) \ \theta + \sum _{1 \le j \le n} (d_j^+ - d_j^-) \alpha _{j} > \alpha _i, $$

in the respective cases. In particular, the subterms of kinds (ii) and (iii) lie in $\widetilde{\textbf{U}}_{d_i \ge 1,+}$. This completes the proof of (5.4).

Let $\mu$ denote the composition of the eastward and southward arrows, and $\nu$ the composition of the southward and eastward arrows in (). We have $\mu (B_0) = \nu (B_0)$ and, by the first part of the proposition, $\mu (B_{-1}) \equiv \nu (B_{-1})$ modulo $U_{d_i=1,+}$. Since $B_0$ and $B_{-1}$ generate $\widetilde{\textbf{U}}^\imath (\widehat{\mathfrak {sl}}_2)$, and $\mu (B_0)$ contains terms of degree $\pm \alpha _i$, it follows that the diagram commutes modulo $\widetilde{\textbf{U}}_{\ne i,+}$. $\square$

## Auxiliary calculations—braid group actions

In Sect. 7, we will express $\textbf{T}_{\omega ^{\prime }_i}(B_i)$ in terms of certain polynomials. The following calculations will be used to prove that those polynomials are *i*-good.

### Lemma 6.1

If $$a_{ji} = -1$$, then:

1. $$[F_j,{\widetilde{E}}_i]_{q_i} = 0$$,
2. $$T_i(F_j) = [F_j, F_i]_{q_i}$$ and $$T_i({\widetilde{E}}_j) = [{\widetilde{E}}_j, {\widetilde{E}}_i]_{q_i}$$.

### Proof

The first part follows from the fact that, by 2.1,

$$\begin{aligned} F_jE_iK_i^{-1}- q_iE_iK_i^{-1}F_j = q_iE_iK_i^{-1}F_j - q_iE_iK_i^{-1}F_j = 0. \end{aligned}$$

In the second part, $T_i(F_j) = [F_j, F_i]_{q_i}$ follows immediately from the definition of the braid group action, and

$$\begin{aligned} T_i({\widetilde{E}}_j) =&\ - q_i^{-2} T_i (\mathbb {K}_j E_j K_j^{-1}) \\ =&\ q_i^{-3} \mathbb {K}_i\mathbb {K}_j (E_jE_i - q_iE_iE_j) K_i^{-1}K_j^{-1}\\ =&\ q_i^{-4} \mathbb {K}_i\mathbb {K}_j (E_jK_j^{-1}E_iK_i^{-1}- q_iE_iK_i^{-1}E_jK_j^{-1}) \\ =&\ [{\widetilde{E}}_j, {\widetilde{E}}_i]_{q_i}. \end{aligned}$$

$$\square $$

### Lemma 6.2

If $$a_{ij}a_{ji} = 2$$, then

$$ \textbf{T}_j \textbf{T}_i (B_j) = \left\{ \begin{array}{r l} \mathbb {K}_i B_j + \sum _{r=0}^2 (-q)^r B_i^{(2-r)} B_j B_i^{(r)} &  \quad \text{ if } \ a_{ji} = -2, \\ {[B_i, B_j]}_{q^2} &  \quad \text{ if } \ a_{ji} = -1. \end{array} \right. $$

### Proof

Let $a_{ji} = -2$. Then, $q_i=q$, $q_j=q^2$ and

$$\begin{aligned} \textbf{T}_i(B_j) = [2]_q^{-1} B_jB_i^2 - qB_iB_jB_i+ q^2 [2]_q^{-1} B_i^2B_j + \mathbb {K}_i B_j. \end{aligned}$$

Moreover, $$T_j( \mathbb {K}_j B_i ) = \mathbb {K}_j B_i$$ and

$$\begin{aligned} \textbf{T}_j( [2]_q^{-1} B_jB_i^2) =&\ [2]_q^{-1} \mathbb {K}_j^{-1} B_jB_iB_jB_iB_j- q^2 [2]_q^{-1} \mathbb {K}_j^{-1}B_jB_iB_j^2B_i \\&- q^2 [2]_q^{-1} \mathbb {K}_j^{-1} B_j^2B_i^2B_j + q^4 [2]_q^{-1} \mathbb {K}_j^{-1} B_j^2B_iB_jB_i, \\ \textbf{T}_j( - qB_iB_jB_i ) =&\ - q \mathbb {K}_j^{-1} B_iB_j^2B_iB_j + q^3 \mathbb {K}_j^{-1} B_iB_j^3B_i \\&+ q^3 \mathbb {K}_j^{-1} B_jB_iB_jB_iB_j- q^5 \mathbb {K}_j^{-1} B_jB_iB_j^2B_i, \\ \textbf{T}_j( q^2 [2]_q^{-1} B_i^2B_j) =&\ q^2 [2]_q^{-1} \mathbb {K}_j^{-1} B_iB_jB_iB_j^2- q^4 [2]_q^{-1} \mathbb {K}_j^{-1} B_iB_j^2B_iB_j \\&- q^4 [2]_q^{-1} \mathbb {K}_j^{-1} B_jB_i^2B_j^2 + q^6 [2]_q^{-1} \mathbb {K}_j^{-1} B_jB_iB_jB_iB_j. \end{aligned}$$

Therefore,

$$\begin{aligned} \textbf{T}_j\textbf{T}_i(B_j) =&\ q \left( q^4 + 1\right) \mathbb {K}_j^{-1} B_jB_iB_jB_iB_j- q^2 \left( q^4 + q^2 + 1\right) [2]_q^{-1} \mathbb {K}_j^{-1} B_jB_iB_j^2B_i \\&- q^2 [2]_q^{-1} \mathbb {K}_j^{-1} B_j^2B_i^2B_j + q^4 [2]_q^{-1} \mathbb {K}_j^{-1} B_j^2B_iB_jB_i \\&- \left( q^4 + q^2 + 1\right) [2]_q^{-1} \mathbb {K}_j^{-1} B_iB_j^2B_iB_j + q^3 \mathbb {K}_j^{-1}B_iB_j^3B_i \\&q^2 [2]_q^{-1} \mathbb {K}_j^{-1} B_iB_jB_iB_j^2 - q^4 [2]_q^{-1} \mathbb {K}_j^{-1} B_jB_i^2B_j^2 + \mathbb {K}_i B_j. \end{aligned}$$

Repeatedly using the relation

$$\begin{aligned} B_j^2 B_i&= (q_j+q_j^{-1}) B_jB_iB_j - B_iB_j^2 - q_j^{-1} \mathbb {K}_j B_i \\&= (q^2+q^{-2}) B_jB_iB_j - B_iB_j^2 - q^{-2} \mathbb {K}_j B_i, \end{aligned}$$

we obtain

$$\begin{aligned}&\textbf{T}_j\textbf{T}_i(B_j) = q^2 [2]_q^{-1} B_jB_i^2 + [2]_q^{-1} B_i^2B_j- qB_iB_jB_i + \mathbb {K}_iB_j. \end{aligned}$$

Next, let $a_{ji}=-1$. Then, $q_i = q^2$ and $q_j = q$. We have $\textbf{T}_i(B_j) = B_jB_i- q^2B_iB_j$ and

$$\begin{aligned} \textbf{T}_j \textbf{T}_i (B_j) =&\ [2]_q^{-1} \mathbb {K}_j^{-1} B_jB_iB_j^2 - q \mathbb {K}_j^{-1} B_j^2B_iB_j + q^2 [2]_q^{-1} \mathbb {K}_j^{-1} B_j^3B_i+ B_jB_i \\&- q^2 [2]_q^{-1} \mathbb {K}_j^{-1} B_i B_j^3 +q^3\mathbb {K}_j^{-1} B_jB_iB_j^2 - q^4 [2]_q^{-1} \mathbb {K}_j^{-1} B_j^2B_i B_j- q^2 B_iB_j \\ =&\ \left( q^4 + q^2 + 1\right) [2]_q^{-1} \mathbb {K}_j^{-1} B_jB_iB_j^2 - \left( q^4 + q^2 + 1\right) [2]_q^{-1} \mathbb {K}_j^{-1} B_j^2B_iB_j \\&+ q^2 [2]_q^{-1} \mathbb {K}_j^{-1} B_j^3B_i + B_jB_i- q^2 [2]_q^{-1} \mathbb {K}_j^{-1} B_iB_j^3- q^2B_iB_j. \end{aligned}$$

Using the Serre-type relation

$$\begin{aligned} B_j^3 B_i =&(q^4 + q^2 + 1) q^{-2} B_j^2B_iB_j- \left( q^4 + q^2 + 1\right) q^{-2} B_jB_iB_j^2 + B_iB_j^3 \\&- [2]_q^2 q^{-1} \mathbb {K}_jB_jB_i + [2]_q^2 q^{-1} \mathbb {K}_jB_iB_j, \end{aligned}$$

it follows that

$$\begin{aligned} \textbf{T}_j \textbf{T}_i (B_j) = [B_i, B_j]_{q^2}, \end{aligned}$$

completing the proof. $\square$

### Lemma 6.3

Let $a_{ij}a_{ji} = 2$. If $a_{ji} = -2$, then

$$\begin{aligned} \mathbb {K}_i {\widetilde{E}}_j + \sum _{r=0}^2 (-q)^r B_i^{(2-r)} {\widetilde{E}}_j B_i^{(r)} = T_jT_i({\widetilde{E}}_j) =&\ \sum _{r=0}^2 (-q)^r {\widetilde{E}}_i^{(2-r)}{\widetilde{E}}_j {\widetilde{E}}_i^{(r)}, \\ T_jT_i(F_j) =&\ \sum _{r=0}^2 (-q)^r F_i^{(2-r)} F_j F_i^{(r)}. \end{aligned}$$

If $$a_{ji} = -1$$ then

$$ [B_i, {\widetilde{E}}_j]_{q^2} = T_jT_i({\widetilde{E}}_j) = [{\widetilde{E}}_i, {\widetilde{E}}_j]_{q^2} , \qquad T_jT_i(F_j) = [F_i, F_j]_{q^2}. $$

### Proof

We prove the $a_{ji} = -2$ case, leaving the other easier case to the reader. We have $q_i = q$ and $q_j = q^2$. First, observe that

6.1 $$\begin{aligned} \sum _{r=0}^2 (-q)^r F_i^{(2-r)} {\widetilde{E}}_j F_i^{(r)} = ([2]^{-1}- q^{-1}+q^{-2}[2]^{-1}) F_i^2 {\widetilde{E}}_j = 0. \end{aligned}$$

Secondly,

6.2 $$\begin{aligned}&[2]^{-1}({\widetilde{E}}_iF_i + F_i{\widetilde{E}}_i){\widetilde{E}}_j - q({\widetilde{E}}_i {\widetilde{E}}_j F_i + F_i{\widetilde{E}}_j {\widetilde{E}}_i) + q^2[2]^{-1}{\widetilde{E}}_j({\widetilde{E}}_iF_i + F_i{\widetilde{E}}_i) \nonumber \\&\quad = [2]^{-1}({\widetilde{E}}_iF_i + F_i{\widetilde{E}}_i){\widetilde{E}}_j - q^{-1}{\widetilde{E}}_iF_i {\widetilde{E}}_j - q^3{\widetilde{E}}_j F_i {\widetilde{E}}_i + q^2[2]^{-1}{\widetilde{E}}_j({\widetilde{E}}_iF_i + F_i{\widetilde{E}}_i) \nonumber \\&\quad =-[2]^{-1}(q^{-2}{\widetilde{E}}_i F_i - F_i{\widetilde{E}}_i) {\widetilde{E}}_j + [2]^{-1}{\widetilde{E}}_j(q^2{\widetilde{E}}_i F_i - q^4 F_i {\widetilde{E}}_i) \nonumber \\&\quad =-q^{-2} [2]^{-1}\mathbb {K}_i\big ( [E_i,F_i] K_i^{-1}{\widetilde{E}}_j - q^4{\widetilde{E}}_j [E_i,F_i] K_i^{-1}\big ) \nonumber \\&\quad = -q^{-2} (q^2 - q^{-2})^{-1}\mathbb {K}_i \big ( (1 - K_i^{-2}){\widetilde{E}}_j - q^4{\widetilde{E}}_j (1 - K_i^{-2}) \big ) \nonumber \\&\quad = -\mathbb {K}_i {\widetilde{E}}_j. \end{aligned}$$

Next, we calculate $T_jT_i({\widetilde{E}}_j)$. By definition,

$$\begin{aligned} T_i(E_j) = [2]^{-1}E_i^2E_j - q^{-1}E_iE_jE_i + q^{-2}[2]^{-1}E_jE_i^2, \end{aligned}$$

and

$$\begin{aligned} T_jT_i(E_j)&= - [2]^{-1}E_jE_iE_jE_iF_jK_j + q^{-2}[2]^{-1}E_jE_i^2E_jF_jK_j \\&\quad + q^{-2}[2]^{-1}E_iE_j^2E_iF_jK_j - q^{-4}[2]^{-1}E_iE_jE_iE_jF_jK_j \\&\quad + q^{-1}E_jE_iF_jK_jE_jE_i - q^{-3} E_jE_iF_jK_jE_iE_j - q^{-3}E_iE_jF_jK_jE_jE_i \\&\quad + q^{-5} E_iE_jF_jK_jE_iE_j - q^{-2}[2]^{-1}F_jK_jE_jE_iE_jE_i\\&\quad + q^{-4}[2]^{-1}F_jK_jE_jE_i^2E_j + q^{-4}[2]^{-1}F_jK_jE_iE_j^2E_i\\&\quad - q^{-6}[2]^{-1}F_jK_jE_iE_jE_iE_j. \end{aligned}$$

Applying relations (2.1)–(2.1), the expression above simplifies to

$$\begin{aligned} T_jT_i(E_j) = - q^{-1}E_iE_jE_i + [2]^{-1}E_jE_i^2 + q^{-2}[2]^{-1}E_i^2E_j. \end{aligned}$$

Since $T_1T_2(\mathbb {K}_1) = \mathbb {K}_2^2 \mathbb {K}_1$ and $T_1T_2(K_1) = K_2^2 K_1$, we obtain

6.3 $$\begin{aligned} \ \ \ T_jT_i({\widetilde{E}}_j)&= q^{-6} \sum _{r=0}^2 (-q)^r E_i^{(2-r)}E_j E_i^{(r)}K_i^{-2}K_j^{-1}\mathbb {K}_i^2 \mathbb {K}_j \nonumber \\  &= \sum _{r=0}^2 (-q)^r {\widetilde{E}}_i^{(2-r)}{\widetilde{E}}_j {\widetilde{E}}_i^{(r)}, \end{aligned}$$

where the second equality follows from the fact that $E_i^{(2-r)}E_j E_i^{(r)}K_i^{-2}K_j^{-1}= (E_iK_i^{-1})^{(2-r)} E_jK_j^{-1}(E_iK_i^{-1})^{(r)}$ for each *r*.

Now, combining (6.1), (6.2) and (6.3), we obtain the first equality in the statement of the lemma. The second equality is obtained via a similar calculation to that of $T_jT_i(E_j)$ above. $\square$

## Weak compatibility

The goal of this section is to prove the following theorem.

### Theorem 7.1

In types $\textsf{A}_n^{(1)}, \textsf{B}_n^{(1)}, \textsf{C}_n^{(1)}, \textsf{D}_n^{(1)}$, for each $1 \le i \le n$, $\textbf{T}_{\omega ^{\prime }_i}(B_i)$ can be expressed as an *i*-good polynomial. Hence,

$$ \eta (\textbf{T}_{\omega _i^{\prime }}(B_i)) \equiv T_{\omega _i^{\prime }}(\eta (B_i)) \mod \widetilde{\textbf{U}}_{d_i \ge 1,+}. $$

The second statement follows immediately from the first by Proposition 5.5. To prove the first statement, we proceed by a direct, case-by-case method. We first explicitly describe the polynomials expressing $\textbf{T}_{\omega ^{\prime }_i}(B_i)$, using the calculations from Sect. 4, and then prove that these polynomials are *i*-good, using the calculations from Sect. 6. Type $\textsf{A}_n^{(1)}$ has already been handled in [32, Proposition 9.6].

### Type $\textsf{D}_n^{(1)}$

For convenience, we will sometimes write $B^{\prime }_l = B_l$ for  and . We use similar notation with *B* replaced by *F*.

#### Weights 

##### Lemma 7.2

Let . If *i* is even, then7.1If *i* is odd, then7.2

##### Proof

Recall the definitions of $\zeta _i$ from Sect. 4 and $\tau _i$ from (5.1). Since $\omega ^{\prime }_i = \zeta _i \tau _i$ and $\ell (\omega ^{\prime }_i) = \ell (\zeta _i) + \ell (\tau _i)$, we have $\textbf{T}_{\omega ^{\prime }_i}(B_i) = \textbf{T}_{\zeta _i} \textbf{T}_{\tau _{i}} (B_{i})$. According to Lemma 5.3 (with  and $l=i$),7.3Using Lemmas 2.4 and 4.2, we observe that applying $\textbf{T}_{\zeta _i}$ to the RHS of (7.3) merely changes the indices of the variables except the last variable . The precise change of the indices is given by Lemma 4.2, andfollows directly from the definition of the braid group action. $\square$

##### Proposition 7.3

The polynomials (7.1)–(7.2) are *i*-good.

##### Proof

We do the even case, the odd one being analogous. First consider the innermost nested polynomial . DefineLet $S_k$ be defined analogously, with each $B_l$ replaced by ${\widetilde{E}}_l$. We will prove by induction that the polynomials $R_k$ are good. Observe that $R_1$ is good trivially, and $R_2$ is good by part (1) of Lemma 6.1. For $k \ge 2$, we haveAccording to the inductive hypothesis,  and $R_k$ are good. Therefore, it suffices to show that $P_2(F^{\prime }_{k+1}, S_k) = 0$. Since $F^{\prime }_{k+1}$ commutes with , Lemma 5.1 implies that7.4which vanishes since $[F^{\prime }_{k+1}, {\widetilde{E}}_k]_q = 0$ by part (1) of Lemma 6.1. We conclude that $R_{k+1}$ is good and, hence, by induction,  is good.

Next, we show that the middle nested polynomial  is good. The proof is similar to the argument above. Setand define $S_k^{\textsf{mid}}$ analogously, with each $B_l$ replaced by ${\widetilde{E}}_l$, and  by . We already know that $R_0^{\textsf{mid}}$ is good. The polynomial $R_1^{\textsf{mid}}$ is also good since, by Lemma 5.2,and  by the same argument as in (7.4).

Next, we claim that  for each . Since , it suffices to show that . This is indeed true becauseTherefore,which implies that $R_{k}^{\textsf{mid}}$ is good. By induction, we conclude that  is good.

Finally, we show that the outer polynomial  is good. Setand define $S_k^{\textsf{out}}$ analogously, with each $B_l$ replaced by ${\widetilde{E}}_l$, and  by . We already know that $R_0^{\textsf{out}}$ is good. The polynomial $R_1^{\textsf{out}}$ is also good sinceand  by part (1) of Lemma 6.1. We also claim that  for each . Since , it suffices to show that . This is indeed true becauseIt follows thatwhich implies that $R_{k}^{\textsf{out}}$ is good. By induction, we conclude that  is good. This proves that (7.1) is a good polynomial.

Next, Lemma 2.4, part (2) of Lemma 5.3, and part (2) of Lemma 6.1 imply that, by uniformly substituting $F_j$ or ${\widetilde{E}}_j$ for each $B_j$ in the proof of Lemma 7.2, we obtain a proof of the following identities:The fact that (7.1) is a good polynomial, together with the two identities above, yields part (2.b) of Definition 5.4 of *i*-goodness. Part (2.a) clearly holds as well, which completes the proof. $\square$

#### Weights $\omega _{n-1}, \omega _n$

We apply similar analysis to weights $\omega _{n-1}$ and $\omega _n$.

##### Proposition 7.4

We have7.57.6The polynomials (7.5)–(7.6) are *i*-good.

##### Proof

Let . Since  and , we have . According to Lemma 5.3 (with  and $l=i$),7.7Using Lemma 4.3, we observe that applying $\textbf{T}_{\zeta _i}$ to the RHS of (7.7) merely shifts the indices of the variables by $-1$, except the last variable $B_{1}$, andThe proof of *i*-goodness follows the same method as the proof of Proposition 7.3, so we omit it. $\square$

### Type $\textsf{B}_n^{(1)}$

In this subsection, only we consider polynomials $P_k(y_1, \cdots , y_k)$ to be defined in the same way as in Sect. 5.1 but with *q* replaced by $q^2$. We will also use the polynomials $\textbf{P}_i(a,b)$ from (5.2).

#### Lemma 7.5

Let . Then,7.8If $i=n$ then

7.9 $$\begin{aligned} \textbf{T}_{\omega ^{\prime }_n}(B_n) = P_n(B_{n-1}, \cdots , B_1, P_n(B_n, \cdots , B_2, B_0)). \end{aligned}$$

#### Proof

Let . Arguing in the same way as in the proof of Lemma 7.2, we find that7.10with $\zeta _i$ given by (4.1). Lemma 4.4 implies that $\textbf{T}_{\zeta _i}$ shifts the indices of all the variables on the RHS of (7.10) by *i* modulo *n*, except the last variable . Moreover,By Lemma 6.2,if *i* is even, with an analogous calculation in the odd case. The proof for $i=n$ is similar to that in type $\textsf{C}_n^{(1)}$, which is done in Lemma 7.7, so we leave it to the reader. $\square$

#### Proposition 7.6

The polynomials (7.8)–(7.9) are *i*-good.

#### Proof

The proof is similar to the proof of Proposition 7.3. Let us explain the differences in the case $1 \le i \le n - 1$. Arguing as in the first paragraph of the proof of Proposition 7.3, we deduce that the polynomials $P_{k}(B_{k}, \cdots , B_2, B_0)$, for $2 \le k \le n-1$, are good. Hence, $S_k:= P_{k}(B_{k}, \cdots , B_2, {\widetilde{E}}_0) = P_{k}({\widetilde{E}}_{k}, \cdots , {\widetilde{E}}_2, {\widetilde{E}}_0)$. Then,

$$\begin{aligned} \textbf{P}_n(S_{n-1}, B_n) = \textbf{P}_n(P_2({\widetilde{E}}_{n-1}, S_{n-2}), B_n) = P_2(\textbf{P}_n({\widetilde{E}}_{n-1}, B_n), S_{n-2}), \end{aligned}$$

and $\textbf{P}({\widetilde{E}}_{n-1}, B_n) = \widehat{\textbf{P}}({\widetilde{E}}_{n-1},{\widetilde{E}}_n)$ by Lemma 6.3. Therefore,

$$ \textbf{P}_n(S_{n-1}, B_n) = P_2(\widehat{\textbf{P}}({\widetilde{E}}_{n-1},{\widetilde{E}}_n), S_{n-2}) = \widehat{\textbf{P}}(S_{n-1}, {\widetilde{E}}_n). $$

The rest of the argument follows the same pattern as in the proof of 7.3. In particular, we obtain7.11Note that (7.11) is indeed a subterm of (7.8) of maximal total degree, and so $P_{--} = T_{\omega ^{\prime }_i}(F_i)$, proving that (7.8) is *i*-good. $\square$

### Type $\textsf{C}_n^{(1)}$

Finally, let us consider type $\textsf{C}_n^{(1)}$.

#### Lemma 7.7

Let $1 \le i \le n - 1$. Then,7.12where $\mathbb {P}(a,b) = [a,b]_{q^2}$. If $i=n$, then

7.13 $$\begin{aligned} \textbf{T}_{\omega ^{\prime }_n}(B_n) = \textbf{P}_{n-1}( \cdots \textbf{P}_2(\textbf{P}_1(B_0, B_1), B_2), \cdots , B_{n-1}). \end{aligned}$$

#### Proof

In the first case, the proof is the same as the proof of Lemma 7.5, with the exception that Lemma 4.5 is used instead of Lemma 4.4, and the case of $a_{ji} = -1$ (rather than $a_{ji} = -2$) from Lemma 6.2 is used.

If $i = n$, then one proceeds as follows. One can rewrite (3.3) as

$$ \omega _n = \pi _n [n,1] \cdots [n,n-1] s_n. $$

By Lemma 6.2, $\textbf{T}_{[n,n-1]}(B_n) = \textbf{P}_{n-1}(B_n,B_{n-1})$. Proceeding by induction, we may assume that

$$ \textbf{T}_{[n,k] \cdots [n,n-1]}(B_n) = \textbf{P}_{k}( \cdots \textbf{P}_{n-2}(\textbf{P}_{n-1}(B_n, B_{n-1}), B_{n-2}), \cdots , B_{k}). $$

By Lemma 2.4, applying $\textbf{T}_{[n,k-1]}$ shifts the indices of all the $B_{l}$ and $\textbf{P}_l$ by $-1$, except for $B_n$, which is transformed into $\textbf{P}_{n-1}(B_n,B_{n-1})$. Finally, $\textbf{T}_{\pi _n}$ reverses the indices. $\square$

#### Proposition 7.8

The polynomials (7.12)–(7.13) are *i*-good.

#### Proof

The proof in the case $1 \le i \le n - 1$ is similar to those in types $\textsf{B}_n^{(1)}$ and $\textsf{D}_n^{(1)}$, so let us consider the case $i = n$. First, observe that an appropriate modification of the proof of Lemma 7.7 yields

7.14 $$\begin{aligned} {T}_{[n,k] \cdots [n,n-1]}({\widetilde{E}}_n) =&\ \widehat{\textbf{P}}( \cdots \widehat{\textbf{P}}(\widehat{\textbf{P}}({\widetilde{E}}_n, {\widetilde{E}}_{n-1}), {\widetilde{E}}_{n-2}), \cdots , {\widetilde{E}}_{k}) =: S_k, \nonumber \\ T_{\omega ^{\prime }_n}({\widetilde{E}}_n) =&\ \widehat{\textbf{P}}( \cdots \widehat{\textbf{P}}(\widehat{\textbf{P}}({\widetilde{E}}_0, {\widetilde{E}}_1), {\widetilde{E}}_2), \cdots , {\widetilde{E}}_{n-1}), \end{aligned}$$

7.15 $$\begin{aligned} T_{\omega ^{\prime }_n}(F_n) =&\ \widehat{\textbf{P}}( \cdots \widehat{\textbf{P}}(\widehat{\textbf{P}}(F_0, F_1), F_2), \cdots , F_{n-1}), \end{aligned}$$

for $$1 \le k \le n-1$$.

We prove by (descending) induction that

7.16 $$\begin{aligned} R_k := \textbf{P}_{k}( \cdots \textbf{P}_{n-2}(\textbf{P}_{n-1}({\widetilde{E}}_n, B_{n-1}), B_{n-2}), \cdots , B_{k}) = S_k. \end{aligned}$$

The base case of $k = n-1$ reduces to Lemma 6.3. Next, suppose that (7.16) holds for *k*. Then,

$$\begin{aligned} R_{k-1} = \textbf{P}_{k-1}(R_k, B_{k-1}) =&\ \textbf{P}_{k-1}(S_k, B_{k-1}) \\ =&\ \mathbb {K}_{k-1} S_k + \sum _{r=0}^2 (-q)^r B_{k-1}^{(2-r)} S_k B_{k-1}^{(r)}. \end{aligned}$$

Let us compute the sum above. Observe that $F_{k-1} S_k = q^2 S_k F_{k-1}$ (since $F_{k-1}$ commutes with all the letters in $S_k$ except for $K_k^{-1}$, which appears twice). Hence,

$$\begin{aligned} \sum _{r=0}^2 (-q)^r F_{k-1}^{(2-r)} S_k F_{k-1}^{(r)} =&\ [2]^{-1}(q^4 - (q+q^{-1})q^3 +q^2)S_k F_{k-1}^2 = 0. \end{aligned}$$

Moreover, an easy calculation shows that

$$\begin{aligned} (F_{k-1}{\widetilde{E}}_{k-1} + {\widetilde{E}}_{k-1} F_{k-1}) S_k \ -&\ q(F_{k-1} S_k {\widetilde{E}}_{k-1} + {\widetilde{E}}_{k-1} S_k F_{k-1}) \\ +&\ q^2 S_k (F_{k-1} {\widetilde{E}}_{k-1} + {\widetilde{E}}_{k-1} F_{k-1}) = - \mathbb {K}_{k-1} S_k. \end{aligned}$$

It follows that $\textbf{P}_{k-1}(S_k, B_{k-1}) = \widehat{\textbf{P}}(S_k, {\widetilde{E}}_{k-1}) = S_{k-1}$, concluding the inductive step.

The induction above yields $R_1 = S_1$. Applying $\textbf{T}_{\pi _n}$, together with (7.14), yields $P_+ = T_{\omega ^{\prime }_i}({\widetilde{E}}_i)$, while (7.15) yields $P_{--} = T_{\omega ^{\prime }_i}(F_i)$. It follows that (7.13) is *i*-good. $\square$

## Strong compatibility

The goal of this section is to prove a generalization of Theorem 2.5 to the classical types $\textsf{B}_n^{(1)}, \textsf{C}_n^{(1)}, \textsf{D}_n^{(1)}$.

### Theorem 8.1

Let $\widehat{{\mathfrak {g}}}$ be of type $\textsf{A}_n^{(1)}, \textsf{B}_n^{(1)}, \textsf{C}_n^{(1)}$ or $\textsf{D}_n^{(1)}$. Then,

8.1 $$\begin{aligned} \eta _{\textbf{s}}(\pmb {\grave{\Theta }}_i(z)) \equiv&\ \xi _{\textbf{s}}(\pmb {\grave{\Theta }}_i(z)) \pmb {\phi }_i^-(z^{-1})\pmb {\phi }_i^+(\mathfrak {C}z) {\mod \widetilde{\textbf{U}}_{+}}[\negthinspace [z]\negthinspace ], \end{aligned}$$

8.2 $$\begin{aligned} \Delta _{\textbf{s}}(\pmb {\grave{\Theta }}_i(z)) \equiv&\ \eta _{\textbf{s}}(\pmb {\grave{\Theta }}_i(z)) \otimes \eta (\pmb {\grave{\Theta }}_i(z)) \quad \mod {\widetilde{\textbf{U}}\otimes \widetilde{\textbf{U}}_{+}}[\negthinspace [z]\negthinspace ]. \end{aligned}$$

Type $\textsf{A}_n^{(1)}$ was already handled in [32, Corollary 9.15]. Here, we will follow the same methodology as in *op. cit.* We begin in Sect. 8.1 by recalling results which generalize to our situation without any extra work.

### Generalized factorization

In this subsection, let $\widehat{{\mathfrak {g}}}$ be of type $\textsf{A}_n^{(1)}, \textsf{B}_n^{(1)}, \textsf{C}_n^{(1)}$ or $\textsf{D}_n^{(1)}$. Set $\mathfrak {C}_i = \mathfrak {C}^{-1}\mathbb {K}_i$.

#### Lemma 8.2

For each $$i \in \mathbb {I}_0$$:

$$ \eta (A_{i,-1}) = x^-_{i,1} - \mathfrak {C}_ix^+_{i,-1}K_i + Q_i, \qquad Q_i \in \widetilde{\textbf{U}}_{d_i \ge 1,+}. $$

Hence,

$$ \eta (A_{i,-1}) \equiv \iota _i \eta (A_{-1}) \quad \mod \widetilde{\textbf{U}}_{d_i \ge 1,+}. $$

Moreover,

$$ \eta (H_{i,1}) \equiv \iota _i\eta (H_1) + q_i^2 \mathfrak {C}_i^{-1}[Q_i, F_i]_{q_i^{-2}} \quad \mod \widetilde{\textbf{U}}_{d_i \ge 2,+}. $$

#### Proof

See [32, Lemmas 9.8–9.9]. $$\square $$

The statement of Lemma 8.2 should also be seen as the definition of the term $Q_i$. As in Sect. 5.2, let us write $\textbf{T}_{\omega ^{\prime }_i}(B_i) = P_+ + P_-$. It then follows from the proof of [32, Lemma 9.8] that, explicitly,

8.3 $$\begin{aligned} Q_i = \mathfrak {C}_i (P_{-} - P_{--}). \end{aligned}$$

#### Proposition 8.3

Let $$r\in {\mathbb Z}$$. If

8.4 $$\begin{aligned} [[Q_i, F_i]_{q_i^{-2}}, x_{i,-r}^-] \in \widetilde{\textbf{U}}_{d_i \ge 1,+}, \end{aligned}$$

then

$$ \eta (A_{i,r}) \equiv \iota _i \eta (A_{r}) \quad \mod \widetilde{\textbf{U}}_{d_i \ge 1,+}. $$

#### Proof

See [32, Proposition 9.10]. $$\square $$

#### Lemma 8.4

If (8.4) is true, then Theorem 8.1 holds.

#### Proof

The proof is the same as in [32, Corollary 9.15]. $\square$

Therefore, it suffices to prove that condition (8.4) holds in each type.

### Explicit computation

Below we calculate explicitly that (8.4) holds in type $\textsf{D}_n^{(1)}$, for weights $\omega _{1}, \cdots , \omega _{n-2}$. The other types and weights can be handled using the same methods.

Given (8.3), an explicit expression for $Q_i$ can be obtained from (7.1)–(7.2).^8^ Substituting either $F_j$ or ${\widetilde{E}}_j$ for each occurrence of a variable $B_j$, we can write $Q_i$ as a sum of polynomials in the variables $F_j$ and ${\widetilde{E}}_j$. In the series of lemmas below, we check that condition (8.4) holds for each of these polynomials. Depending on the type of polynomial *P*, we prove one of the following: (i) *P* vanishes, (ii) $[P, F_i]_{q^{-2}} \in \widetilde{\textbf{U}}_{d_i \ge 2,+}$, and (iii) *P* can, modulo $\widetilde{\textbf{U}}_{d_i \ge 2,+}$, be expressed as a polynomials in $x_{j,r}^+$ $(j \ne i)$.

Let ${\tilde{e}}_i^+ = {\widetilde{E}}_i$ and ${\tilde{e}}_i^- = F_i$. Let us also abbreviate

#### Lemma 8.5

If $$2 \le i \le n-2$$, then

#### Proof

We calculateFor degree reasons, we only need to check that the commutator of $F_i$ withvanishes. This reduces to an easily verifiable calculation in the finite quantum group of type $\textsf{A}_3$. $\square$

#### Lemma 8.6

If $$1 \le i \le n-2$$, then8.58.6

#### Proof

By [32, Lemmas 9.3-$-$9.4], the LHS of (8.5) reduces toThe lemma now follows from the following two equalities8.78.8which can be checked directly.^9^ (They reduce to calculations in finite quantum groups of type $\textsf{A}_3$ and $\textsf{D}_4$, respectively.) $\square$

#### Lemma 8.7

Let $2 \le i \le n-2$ andThe element $[Z, F_i]_{q^{-2}}$ can be expressed as a polynomial in $x_{j,r}^+$ $(j \ne i)$, with coefficients in ${\mathbb C}$. Hence $[[Z, F_i]_{q^{-2}}, x_{i,s}^-] =0$, for all $s \in {\mathbb Z}$.

#### Proof

The proof is analogous to [32, Lemmas 9.13]. $\square$

Let

#### Lemma 8.8

Let $$1 \le i \le n-2$$. Then,

#### Proof

This follows from (8.7)–(8.8). $\square$

#### Lemma 8.9

The element $[Z^{\prime }, F_i]_{q^{-2}}$ can be expressed as a polynomials in $x_{j,r}^+$ $(j \ne i)$, with coefficients in ${\mathbb C}$, modulo $\widetilde{\textbf{U}}_{d_i \ge 2,+}$. Hence, $[[Z^{\prime }, F_i]_{q^{-2}}, x_{i,s}^-] = 0$ modulo $\widetilde{\textbf{U}}_{d_i \ge 1,+}$, for all $s \in {\mathbb Z}$. The same holds if $Z^{\prime }$ is replaced by (8.6).

#### Proof

The proof is analogous to [32, Lemmas 9.13]. $\square$

## Application to *q*-characters

We propose a generalization of the notion of *q*-characters to affine quantum symmetric pairs, based on the Lu–Wang Drinfeld-type presentation. We apply Theorem 8.1 to show that our construction is compatible with the usual *q*-character map for quantum affine algebras.

### *q*-characters of quantum affine algebras

Fix $\textbf{c} = (c_0, \cdots , c_n) \in ({\mathbb C}^\times )^{n+1}$ and let *C* be the image of $\mathfrak {C}$ in $\textbf{U}^\imath _{\textbf{c}}$. Let $\operatorname {Rep}\textbf{U}$ be the monoidal category of finite-dimensional representations of $\textbf{U}$. It acts on $\operatorname {Rep}\textbf{U}^\imath _{\textbf{c}}$, the category of finite-dimensional representations of $\textbf{U}^\imath _{\textbf{c}}$, via the coproduct $\Delta _{\textbf{c},\textbf{s}}$ (2.5). It follows from [15, Sec. 3.5] (see also [32, Lemmas 2.4]) that any such monoidal action is, up to twisting by a character, equal to the “standard” monoidal action with $\textbf{s} = (0, \cdots , 0)$. Therefore, henceforth we shall assume that we are working with the standard monoidal action.

We will use the same notations for the Grothendieck groups of the categories above. From this point of view, $\operatorname {Rep}\textbf{U}$ is a ring acting on $\operatorname {Rep}\textbf{U}^\imath _{\textbf{c}}$. Let $U_q(\widetilde{\mathfrak {h}})$ be the subalgebra of $\textbf{U}$ generated by $h_{i,k}$ ($i \in \mathbb {I}_0$, $k < 0$). Following [17, 19], set

9.1 $$\begin{aligned} Y_{i,a} =&\ K_{\omega _i}^{-1} \exp \left( -(q-q^{-1}) \sum _{k > 0} {\tilde{h}}_{i,-k} a^k z^k \right) \in {U_q(\widetilde{\mathfrak {h}})}[\negthinspace [z]\negthinspace ]\qquad (a \in {\mathbb C}^\times ), \end{aligned}$$

where

$$\begin{aligned} {\tilde{h}}_{i,-k} = \sum _{j \in \mathbb {I}_0} {\widetilde{C}}_{ji}(q^k) h_{j,-k}, \end{aligned}$$

and ${\widetilde{C}}(q)$ is the inverse of the *q*-Cartan matrix. Let $\mathcal {Y}= {\mathbb Z}[Y_{i,a}^{\pm 1}]_{i \in \mathbb {I}_0, a \in {\mathbb C}^{\times }}$. By [19, Theorem 3], there exists an injective ring homomorphism $\chi _q:\operatorname {Rep}\textbf{U}\rightarrow \mathcal {Y}\subset {U_q(\widetilde{\mathfrak {h}})}[\negthinspace [z]\negthinspace ]$, called the *q-character map*, given by

$$ [V] \mapsto \operatorname {Tr}_V \left[ \!\exp \left( \! -(q-q^{-1}) \sum _{i \in \mathbb {I}_0} \sum _{k >0} \frac{k}{[k]_{q_i}} \pi _V(h_{i,k}) \otimes {\tilde{h}}_{i,-k} z^k\! \right) \cdot (\pi _V \otimes 1)(T) \!\right] , $$

where *T* is as in [19, (3.8)], and $\pi _V :\textbf{U}\rightarrow \operatorname {End}(V)$ is the representation.

The expression above derives from the Khoroshkin–Tolstoy–Levendorsky–Soibelman–Stukopkin–Damiani (KTLSSD) factorization of the universal *R*-matrix [12, 23, 26]. However, by [17, Proposition 2.4], the *q*-character map can equivalently be defined more explicitly in terms of the joint spectrum of the Drinfeld–Cartan operators (i.e., the coefficients of the series $\pmb {\phi }^{\pm }_i(z)$). More precisely, there is a one-to-one correspondence between the monomials occurring in $\chi _q(V)$ and the common eigenvalues of $\pmb {\phi }^{\pm }_i(z)$ on *V*. Let $V = \bigoplus _{\gamma } V_{\gamma }$, with $\gamma = (\gamma ^\pm _{i,\pm m})_{i \in \mathbb {I}_0, m \in {\mathbb Z}_{\ge 0}}$, where

$$\begin{aligned} V_{\gamma } = \{ v \in V \mid \exists p \ \forall i \in \mathbb {I}_0 \ \forall m \in {\mathbb Z}_{\ge 0}: (\psi ^\pm _{i,\pm m} - \gamma ^\pm _{i,\pm m})^p \cdot v = 0 \}, \end{aligned}$$

be the generalized eigenspace decomposition of *V*. Collect the eigenvalues into generating series $\gamma ^\pm _i(z) = \sum _{m \ge 0} \gamma ^\pm _{i,\pm m} z^{\pm m}$. By [17, Proposition 2.4], the series $\gamma ^\pm _i(z)$ are expansions (at 0 and $\infty$, respectively) of the same rational function of the form

$$ q_i^{\deg Q_i - \deg R_i} \frac{Q_i(q_i^{-1}z)R_i(q_iz)}{Q_i(q_iz)R_i(q_i^{-1}z)}, $$

for some polynomials $Q_i(z), R_i(z)$ with constant term 1. Writing

9.2 $$\begin{aligned} Q_i(z) = \prod _{r=1}^{k_i} (1 - z a_{i,r}), \qquad R_i(z) = \prod _{s=1}^{l_i} (1 - z b_{i,s}), \end{aligned}$$

the *q*-character of *V* can now be expressed as

$$ \chi _q(V) = \sum _{\gamma } \dim (V_\gamma ) M_\gamma , \qquad M_\gamma = \prod _{i \in \mathbb {I}_0} \prod _{r=1}^{k_i} Y_{i,a_{i,r}} \prod _{s=1}^{l_i} Y_{i,b_{i,s}}^{-1}. $$

### Boundary *q*-characters

The notion of a quasi-*K*-matrix appeared first in the work of Bao and Wang [8, 9] on canonical bases for quantum symmetric pairs, as an intertwiner between the bar involutions on the quantum group and the coideal subalgebra. This construction of the quasi-*K*-matrix was later extended to arbitrary Kac–Moody type by Balagović and Kolb in [5] and, in finite type, led to the realization of the universal *K*-matrix as a coideal intertwiner [5, 9]. Later, Appel and Vlaar [2, 3] generalized these methods to obtain a universal *K*-matrix in the affine case. However, a KTLSSD-type factorization is not yet available.^10^ Therefore, we propose to initiate the study of *q*-characters for affine quantum symmetric pairs based on the Lu–Wang (Drinfeld-type) presentation.

#### Definition 9.1

Let

$$\begin{aligned} \mathcal {K}^0&= \frac{1-q_i^{-2}C z^2}{1-C z^2} \exp \left( -(q-q^{-1}) \sum _{i \in \mathbb {I}_0} \sum _{k >0} \frac{k}{[k]_{q_i}} H_{i,k} \otimes {\tilde{h}}_{i,-k} z^k \right) \\&\quad \in {\textbf{U}^\imath _{\textbf{c}}\otimes U_q(\widetilde{\mathfrak {h}})}[\negthinspace [z]\negthinspace ]. \end{aligned}$$

We define the *boundary* *q-character map* to be

$$ \chi _q^\imath :\operatorname {Rep}\textbf{U}^\imath _{\textbf{c}}\ \rightarrow \ {U_q(\widetilde{\mathfrak {h}})}[\negthinspace [z]\negthinspace ], \qquad [V] \mapsto \operatorname {Tr}_V(\mathcal {K}^0 \circ (\pi _V \otimes 1)). $$

Consider ${U_q(\widetilde{\mathfrak {h}})}[\negthinspace [z]\negthinspace ]$ as a $\mathcal {Y}$-module via the ring homomorphism

9.3 $$\begin{aligned} \mathcal {Y}\rightarrow \mathcal {Y}\hookrightarrow {U_q(\widetilde{\mathfrak {h}})}[\negthinspace [z]\negthinspace ], \qquad Y_{i,a} \mapsto Y_{i,Ca}Y_{i,a^{-1}}^{-1}. \end{aligned}$$

Below we apply Theorem 8.1 to show that our boundary *q*-character map is a module homomorphism. First, we need some notation. Given a polynomial $P(z) \in {\mathbb C}[z]$ with constant term 1, let $P^\dag (z)$ be the polynomial with constant term 1 whose roots are obtained from those of *P*(*z*) via the transformation $a \mapsto C^{-1}a^{-1}$, and let $P^*(z)$ be the polynomial with constant term 1 whose roots are the inverses of the roots of *P*(*z*).

#### Proposition 9.2

Let $W \in \operatorname {Rep}\textbf{U}$. Then, the generalized eigenvalues of $\pmb {\grave{\Theta }}_i(z)$ on the restricted representation $\eta _{\textbf{c}}^*(W)$ are of the form:

$$\begin{aligned} \gamma _i^\iota (z) = \frac{\textbf{Q}_i(q_i^{-1}z)}{\textbf{Q}_i(q_i z)} \frac{\textbf{Q}_i^\dag (q_i z)}{\textbf{Q}_i^\dag (q_i^{-1}z)}, \end{aligned}$$

where $\textbf{Q}_i(z)$ is a polynomial with constant term 1. Explicitly,

$$ \textbf{Q}_i(z) = {Q_i(Cz)R_i^*(z)}, \quad \textbf{Q}_i^\dag (z) = {R_i(Cz)Q_i^*(z)}. $$

#### Proof

Theorem 8.1 implies that the action of $\pmb {\grave{\Theta }}_i(z)$ on $\eta _{\textbf{c}}^*(W)$ is the sum of the action of $\pmb {\phi }_i^-(z^{-1})\pmb {\phi }_i^+(C z)$ and a nilpotent operator. Hence, the eigenvalues of $\pmb {\grave{\Theta }}_i(z)$ coincide with those of $\pmb {\phi }_i^-(z^{-1})\pmb {\phi }_i^+(C z)$. The result now follows from [19, Proposition 1] by the same argument as in the proof of [32, Corollary 5.1]. $\square$

#### Corollary 9.3

Let $(\textbf{U}, \textbf{U}^\imath _{\textbf{c}})$ be a split quantum symmetric pair of type $\textsf{A}_n^{(1)}, \textsf{B}_n^{(1)}, \textsf{C}_n^{(1)}$ or $\textsf{D}_n^{(1)}$. Then, the following diagram commutes: 
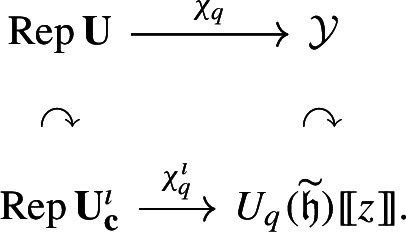


#### Proof

The proof is a modification of the proof of [17, Proposition 2.4]. Let $W \in \operatorname {Rep}\textbf{U}$ and $V \in \operatorname {Rep}\textbf{U}^\imath _{\textbf{c}}$. We need to calculate $\chi _q^\imath (V \otimes W)$. Formulae (8.1)–(8.2) in Theorem 8.1, together with Proposition 9.2, imply that the eigenvalues of $\pmb {\grave{\Theta }}_i(z)$ on $V \otimes W$ are a product of the eigenvalues of $\pmb {\grave{\Theta }}_i(z)$ on *V*, and $\gamma _i^\iota (z)$. Hence, $\operatorname {Tr}_{V\otimes W}(\mathcal {K}^0 \circ (\pi _{V \otimes W} \otimes 1)) = \operatorname {Tr}_V(\mathcal {K}^0 \circ (\pi _V \otimes 1)) \cdot \operatorname {Tr}_W(\mathcal {K}^0 \circ (\pi _W \otimes 1))$, and the eigenvalues of $H_{i,m}$ on *W* are (up to the overall normalization factor $\frac{1-q_i^{-2}Cz^2}{1-Cz^2}$) of the form

9.4 $$\begin{aligned} \frac{q_i^m - q_i^{-m}}{n(q-q^{-1})}\left( \sum _{r=1}^{k_i} ((Ca_{i,r})^m - a_{i,r}^{-m}) - \sum _{s=1}^{l_i} ((Cb_{i,s})^m - b_{i,s}^{-m}) \right) , \end{aligned}$$

keeping the notation from (9.2). Plugging (9.4) into the definition of $\chi _q^\imath (W)$ and comparing with (9.1), we conclude that $\chi _q^\imath (W)$ is a linear combination of monomials of the form

$$ \prod _{i \in \mathbb {I}_0} \prod _{r=1}^{k_i} Y_{i,C a_{i,r}} Y_{i,a_{i,r}}^{-1}\prod _{s=1}^{l_i} Y_{i, b_{i,s}} Y_{i,C b_{i,s}}^{-1}. $$

Therefore, $\chi _q^\imath (W)$ is equal to the image of $\chi _q(W)$ under (9.3), completing the proof. $\square$

#### Remark 9.4

In the case of the ordinary *q*-character map $\chi _q$, the property of being a ring homomorphism follows almost directly from its definition via the universal *R*-matrix (and the associated properties of transfer matrices). In contrast, in our case, the fact that $\chi _q^\imath$ intertwines the actions of $\operatorname {Rep}\textbf{U}$ and $\mathcal {Y}$ is non-trivial, since we are not using a universal *K*-matrix, but working with the Drinfeld-type presentation instead.

Finally, let us consider the rank one example of the *q*-Onsager algebra.

#### Example 9.5

We calculate the boundary *q*-characters of restricted evaluation representations, for $(s_0,s_1) = (0,0)$. Let $W_n(a)$ be the irreducible $n+1$-dimensional representation of $U_q(\mathfrak {sl}_2)$ evaluated at *qa*. Then, by [19, (4.3)], its *q*-character is

$$ \chi _q(W_n(a)) = \sum _{i=0}^n M_i, \qquad M_i = \prod _{k=i+1}^n Y_{aq^{n-2k+1}} \prod _{k=1}^i Y_{aq^{n-2k+3}}^{-1}. $$

By Corollary 9.3,

$$\begin{aligned} \chi _q^\imath (W_n(a))&= \sum _{i=0}^n \textbf{M}_i,\\&\textbf{M}_i= \prod _{k=i+1}^n Y_{Caq^{n-2k+1}}Y_{a^{-1}q^{-n+2k-1}}^{-1}\prod _{k=1}^i Y_{a^{-1}q^{-n+2k-3}} Y_{Caq^{n-2k+3}}^{-1}. \end{aligned}$$

Writing $\chi _q(W_n(q^{-2}C^{-1}a^{-1})) = \sum _{i=0}^n M^{\prime }_i$ and $\chi _q^\imath (W_n(q^{-2}C^{-1}a^{-1})) = \sum _{i=0}^n \textbf{M}^{\prime }_i$, it is easy to see that $\textbf{M}_i = \textbf{M}^{\prime }_{n-i}$, which implies that

$$ \chi _q^\imath (W_n(a)) = \chi _q^\imath (W_n(q^{-2}C^{-1}a^{-1})). $$

This is not a coincidence since, by [21, Theorem 1.17], $W_n(a)$ and $W_n(q^{-2}C^{-1}a^{-1})$ are indeed isomorphic as representations of the *q*-Onsager algebra.

## Data Availability

The authors declare that all the data supporting the findings of this article are available within the paper.

## Footnotes

1 A recursive formula can be found in [27].

2 One has $\pmb {\grave{\Theta }}_i(z) = (q_i-q_i^{-1}) \acute{\Theta }_i(z)$, where $\acute{\Theta }_i(z) = \sum _{r\ge 0} \acute{\Theta }_{i,r} z^r$ is the series from [29, (2.10)], whose coefficients are the imaginary root vectors from [6], rescaled by $-q_i^{-2}$.

3 If $i=n$, the expression below reads $\pi _1 [1,n] \big (s_0[2,n][1,n]\big )^{\frac{n-1}{2}}$.

4 If $i=n$, the expression below reads $\big (s_0[2,n][1,n]\big )^{\frac{n}{2}}$.

5 For example, suppose that $P(B_0, \cdots , B_n) = B_2^2B_1$. Then,

$$ B_2^2B_1 = {\widetilde{E}}^2_2{\widetilde{E}}_1 + {\widetilde{E}}_2^2 F_1 + ({\widetilde{E}}_2F_2{\widetilde{E}}_1 + F_2 {\widetilde{E}}_2 {\widetilde{E}}_1) +( {\widetilde{E}}_2F_2F_1 + F_2 {\widetilde{E}}_2 F_1) + F_2^2{\widetilde{E}}_1 + F_2^2F_1, $$

i.e., the monomial $B_2^2B_1$ becomes a sum of six homogeneous polynomials.

6 For example, for $P_{\underline{d}}(\cdot ) = {\widetilde{E}}_2^2F_1$, one has $d_2^+ = 2$, $d_1^- = 1$.

7 The inequality below should be read as a family of inequalities of the coefficients along each simple root.

8 For simplicity, we use formula (7.1). In the odd case, indices *n* and $n-1$ should be switched.

9 Such equalities can easily be verified using, e.g., GAP’s QuaGroup Package [14]. For an explicit code, see https://github.com/lijr07/identities_in_quantum_groups.

10 In the finite case, a factorization of quasi *K*-matrices was established in [15, 33].
